# Pomalidomide restores immune recognition of primary effusion lymphoma through upregulation of ICAM-1 and B7-2

**DOI:** 10.1371/journal.ppat.1009091

**Published:** 2021-01-07

**Authors:** Prabha Shrestha, David A. Davis, Hannah K. Jaeger, Alexandra Stream, Ashley I. Aisabor, Robert Yarchoan

**Affiliations:** HIV and AIDS Malignancy Branch, Center for Cancer Research, National Cancer Institute, Bethesda, Maryland, United States of America; Wistar Institute, UNITED STATES

## Abstract

Pomalidomide (Pom) is an immunomodulatory drug that has efficacy against Kaposi’s sarcoma, a tumor caused by Kaposi’s sarcoma-associated herpesvirus (KSHV). Pom also induces direct cytotoxicity in primary effusion lymphoma (PEL), a B-cell malignancy caused by KSHV, in part through downregulation of IRF4, cMyc, and CK1α as a result of its interaction with cereblon, a cellular E3 ubiquitin ligase. Additionally, Pom can reverse KSHV-induced downregulation of MHCI and co-stimulatory immune surface molecules ICAM-1 and B7-2 on PELs. Here, we show for the first time that Pom-induced increases in ICAM-1 and B7-2 on PEL cells lead to an increase in both T-cell activation and NK-mediated cytotoxicity against PEL. The increase in T-cell activation can be prevented by blocking ICAM-1 and/or B7-2 on the PEL cell surface, suggesting that both ICAM-1 and B7-2 are important for T-cell co-stimulation by PELs. To gain mechanistic insights into Pom’s effects on surface markers, we generated Pom-resistant (PomR) PEL cells, which showed about 90% reduction in cereblon protein level and only minimal changes in IRF4 and cMyc upon Pom treatment. Pom no longer upregulated ICAM-1 and B7-2 on the surface of PomR cells, nor did it increase T-cell and NK-cell activation. Cereblon-knockout cells behaved similarly to the pomR cells upon Pom-treatment, suggesting that Pom’s interaction with cereblon is necessary for these effects. Further mechanistic studies revealed PI3K signaling pathway as being important for Pom-induced increases in these molecules. These observations provide a rationale for the study of Pom as therapy in treating PEL and other KSHV-associated tumors.

## Author summary

Primary effusion lymphoma (PEL) is an aggressive B-cell lymphoma caused by Kaposi’s sarcoma-associated herpesvirus (KSHV). KSHV encodes various genes that enable infected cells to evade recognition and elimination by the immune system. PEL cells are poorly recognized by T-cells and NK cells, partly due to KSHV-induced downregulation of immune stimulatory surface molecules ICAM-1 and B7-2. We previously found that a cereblon-binding immunomodulatory drug pomalidomide (Pom) can restore the levels of these markers on PELs. Here, we show that the increases in ICAM-1 and B7-2 induced by Pom leads to a functional increase in the recognition and killing of PELs by both T-cells and NK cells. Further, exposure of both the PEL cells and T-cells to Pom lead to an even higher T-cell stimulation providing strong evidence that Pom could help PEL patients by providing specific immune-stimulatory effect. We further perform mechanistic studies and show that Pom’s cellular binding partner cereblon as well as the PI3K pathway are important for Pom-mediated increases in these surface markers.

## Introduction

Primary effusion lymphoma (PEL) is a rare but aggressive non-Hodgkin’s B cell lymphoma that occurs primarily in patients with human immunodeficiency virus (HIV) infection. PEL is usually treated with combination chemotherapy; however, the overall median survival rate remains approximately two years [1], highlighting the need for new therapeutic modalities for this disease. PEL is caused by Kaposi’s sarcoma-associated herpesvirus (KSHV), which also causes two other B-cell hyperproliferative disorders, KSHV-associated multicentric Castleman’s disease (MCD) and KSHV-associated inflammatory cytokine syndrome (KICS), as well as Kaposi’s sarcoma (KS), an endothelial cell malignancy [2]. Like other herpesviruses, KSHV establishes life-long infection in its hosts, mostly as a latent virus that can be reactivated to produce virions upon exposure to various stimuli. Like most other chronic viruses, KSHV uses various strategies to evade the immune system. One of these strategies is the inhibition of expression of immune surface markers on infected cells, which is accomplished through several mechanisms. Multiple KSHV genes can block type I interferon (IFN), leading to reduced transcription of various genes in the antigen-presentation pathway, including major histocompatibility antigen class I (MHCI) and class II (MHCII) (reviewed in [3]). Latency-associated nuclear antigen-1 (LANA-1) can inhibit MHCI-peptide transport to the membrane by blocking transporter associated with antigen processing (TAP1) [4]. Two KSHV lytic genes, K3 and K5, are E3 ubiquitin ligases that can degrade various cell surface immune molecules including MHCI, intracellular adhesion molecule 1 (ICAM-1/CD54), and B7-2 (CD86) [5–8]. Consistent with these observations, *de novo* infection of primary B cells with KSHV leads to a decrease in surface ICAM-1 and B7-2 [9]. Moreover, PEL cell lines that express lower levels of ICAM-1 and lymphocyte function-associated antigen 3 (LFA-3/CD58) are poorly recognized by T-cells and lymphokine-activated killer (LAK) cells [10]. These observations underscore the role of immune marker suppression as an important immune evasion strategy for the virus and further suggest that restoration of these markers could potentially enhance the recognition and elimination of KSHV-infected tumor cells by the immune system.

Recent *in vitro* and clinical studies have shown that thalidomide (Thal) and related immunomodulatory drugs, particularly Thal-analogs lenalidomide (Len) and pomalidomide (Pom), could benefit patients with KSHV-associated diseases through anti-tumor and immunomodulatory effects. These drugs are approved by the United States Food and Drug Administration (FDA) for use in multiple myeloma (MM), myelodysplastic syndrome (MDS), and erythema nodosum leprosum, and they are in clinical trials for use in various other hematological cancers as well as autoimmune diseases. These three drugs have all been shown to have activity in patients with KS [11–13], and Pom was recently granted accelerated approval by the FDA for use in KS in both HIV-negative patients and HIV-infected patients. In a recent case report, Len was shown to effectively treat a patient with PEL [14].

The primary cellular target of all these drugs is cereblon, a cellular E3 ubiquitin ligase [15], and the drugs will be referred to here as cereblon-binding immunomodulators (CBIs). CBIs bind to and alter the substrate specificity of cereblon, which leads to degradation of transcription factors such as IKZF1(Ikaros), IKZF3(Aiolos), and casein kinase 1 alpha (CK1α), as well as downregulation of interferon regulatory factor 4 (IRF4) and cMyc [16–18]. Although the exact mechanisms of action for these drugs in various diseases, including in MM and KS, aren’t fully understood, many of their immunologic activities are thought to be associated with their ability to regulate cytokine production by the immune cells and/or the tumor microenvironment. CBIs are also able to increase the co-stimulation of both CD4^+^ and CD8^+^ T-cells by enhancing the AP-1 and NFkB transcriptional activity downstream of the CD28 signaling pathway, resulting in increased production of IFN-gamma and IL-2 by the T-cells [19–22]. These cytokines, in addition to direct activity of CBIs on the natural killer cells (NK-cells), can enhance NK-cell responses against the tumor cells in an antibody-independent process [19,23]. CBIs can also exert direct effects on the tumor cells. *In vitro* and *in vivo* data have shown that CBIs can cause direct cytotoxicity in PEL cell lines by causing G0/G1 cell cycle arrest [24]. The direct cytotoxic effects of CBIs in MM and PEL cells occur through cereblon-dependent downregulation of IRF4, cMyc, and to some degree, CK1α [17,18,24,25]. In addition, CBIs have been reported to increase the expression of various immune surface markers such as NK-cell activating ligands MICA and PVR/CD155 in multiple myeloma cells [26], and T-cell co-stimulatory molecules CD80 (B7-1) and CD86 (B7-2) in leukemic B cells of chronic lymphocytic leukemia (CLL) [27].

PEL cell lines, which are latently infected with KSHV, generally express very low levels of ICAM-1 and B7-2 and show a significant downregulation of MHCI during lytic reactivation, rendering these cells relatively invisible to the immune system. Our group recently found that Pom can increase the surface expression of MHCI, ICAM-1, and B7-2 in PEL cell lines, as well as certain tumor lines infected with Epstein-Barr virus (EBV), or human T-cell leukemia virus-1 (HTLV-1) [28,29]. In the current study, we sought to determine whether these increases lead to enhanced recognition of PEL cells by the immune effector cells and to understand the mechanism(s) by which Pom can cause the upregulation of B7-2 and ICAM-1 in PEL cells.

## Results

### PEL cell lines elicit a poor T-cell response

PEL cell lines elicit poor T-cell responses compared to those infected with EBV [30]. This difference has been attributed to the relatively low levels of surface MHCI molecules and a deficient assembly of the MHCI-antigen complex, partly due to lower levels of TAP1 expression in PEL cell lines [30]. The activation of T-cells requires a “two signal” activation process, which involves the stimulation of CD3/TCR signaling by MHC-bound antigen as well as co-stimulatory signals provided by the engagement of CD28, LFA-1, and other receptors on T-cells by their respective ligands [31]. B7-2 and ICAM-1 are the primary activating ligands for CD28 and LFA-1, respectively [32,33]. Therefore, we analyzed the surface expression levels of ICAM-1 and B7-2 in two KSHV singly positive PEL cell lines, BCBL-1 and BC-3, one KSHV and EBV dual positive PEL cell line, JSC-1, two EBV-infected Burkitt’s lymphoma (BL) cell lines, Raji and Daudi, and a virus-negative B cell lymphoma line, MC116 (S1 Fig). The levels of both ICAM-1 and B7-2 were highest in the uninfected MC116 line, with the BL lines expressing moderate levels and the PEL lines expressing the lowest, almost undetectable levels of these markers. We then measured the ability of these lines to elicit T-cell responses using a Jurkat leukemic T-cell line engineered to express luciferase under control of the IL-2 promoter (IL2-Jurkat) as the effector cells. The Jurkat is a useful model system for studying early events in the T-cell activation process [34]. It can be activated through CD3/TCR stimulation and additionally can be costimulated by B7-2/CD28 interaction in a manner that mimics the physiological “two signal” activation process of primary T-cells [34,35]. Further, it expresses IL-2 as an early cytokine response upon activation, and the engineered line expresses luciferase, upon activation [34]. We activated the TCR/CD3 signaling in the IL-2 Jurkat cells using OKT3 anti-human CD3 antibody and co-incubated them for 6 hours with various lymphoma lines as the source for co-stimulatory signals. The T-cell responses against the lymphomas generally tracked with the relative levels of ICAM-1 and B7-2 on the cell surface of these lines (S1A and S1B Fig). All three PEL lines tested showed substantially lower T-cell activation compared to MC116 and BL lines (S1 Fig), suggesting that the low T-cell responses against PELs may be due to low ICAM-1 and B7-2 levels on the PEL cell surface.

### Pom-treatment of PELs lead to an increase in Jurkat T-cell activation

**Fig 1:**
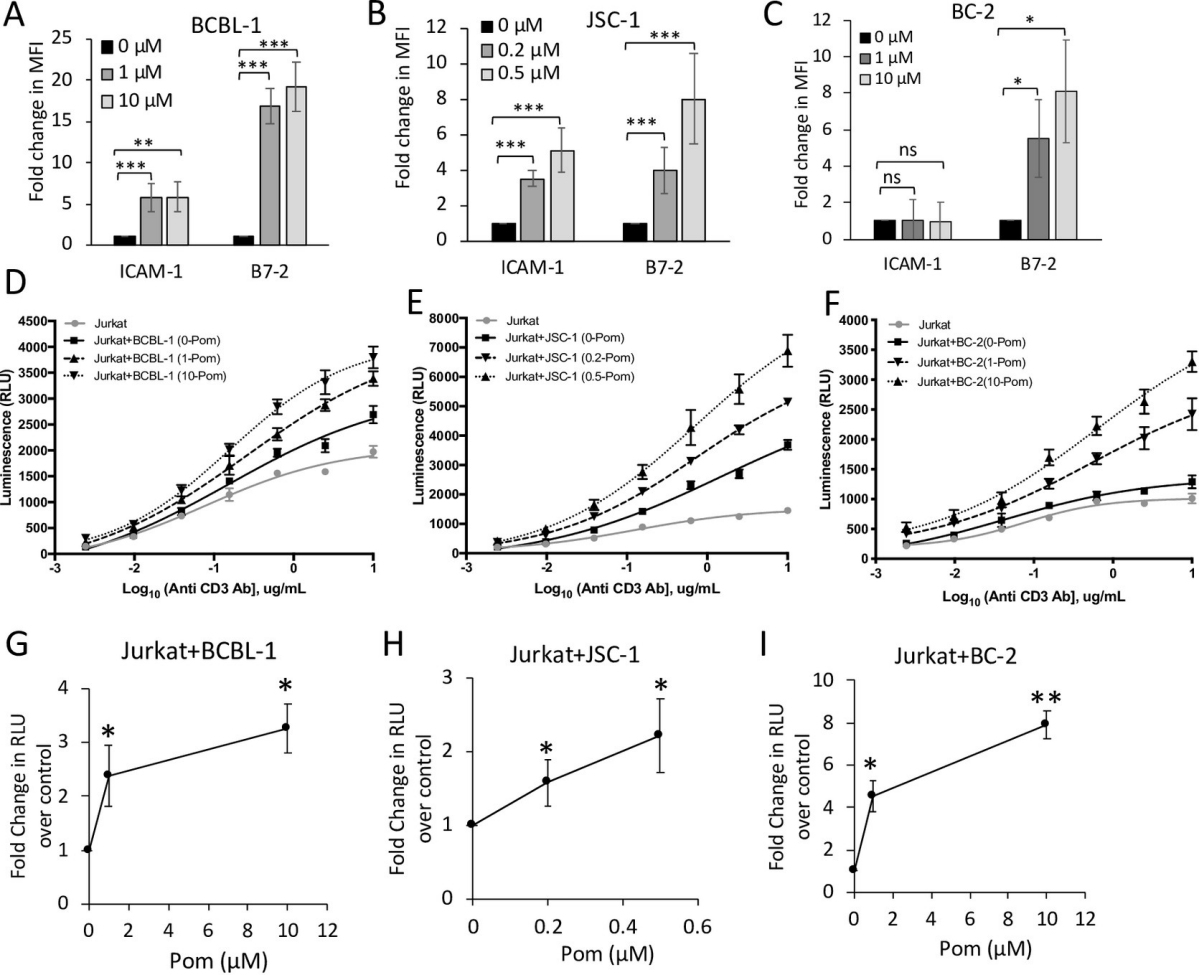
Pom-treated PEL cells express increased ICAM-1 and B7-2 and induce increased T-cell activation. (A—C) Surface expression of ICAM-1 and B7-2 in BCBL-1(A), JSC-1(B), and BC-2(C) cells treated with indicated concentrations of Pom for 48 hours (BCBL-1 and JSC-1) or 72 hours (BC-2). Cells were stained with PerCP/Cy5.5-conjugated IgG isotype control, anti-ICAM-1, or anti-B7-2 antibodies and analyzed using flow cytometry. Results are expressed as average fold change in median fluorescent intensity (MFI) in Pom-treated cells over non-Pom-treated control cells from at least 3 experiments (6 for BCBL-1, 8 for JSC-1, and 3 for BC-2); error bars indicate the standard deviations. (D—I) PEL cells were grown in the presence of DMSO ctrl (0 Pom) or indicated concentrations of Pom. After 48 hours (BCBL-1 and JSC-1) or 72 hours (BC-2), PEL cells were washed with PBS to remove Pom and were coincubated for 6 hours with IL2-Jurkat cells at a 2:1 target to effector ratio in the presence of anti-human CD3 antibody to measure Jurkat T-cell activation. Note that Jurkat T-cells were never exposed to Pom in these experiments. (D–F) Activation of IL2-Jurkat T-cells in the absence or presence of Pom-treated or control BCBL-1(D), JSC-1(E), and BC-2(F) cells expressed as relative light units (RLU). (G–I) Average fold changes in RLU from IL2-Jurkat cells coincubated with Pom-treated BCBL-1, JSC-1, and BC-2 cells over those coincubated with DMSO-ctrl (0μM Pom) treated cells in the presence of 2.5μg/mL anti CD3 antibody. Error bars represent standard deviations from 3 independent experiments. Statistically significant differences (*p≤0.05, **p≤0.01, and ***p≤0.001, ns not significant paired 2-tailed *t*-test) between control and Pom-treated cells are indicated.

We previously found that Pom can increase the surface expression of ICAM-1 and B7-2 in the BCBL-1, JSC-1, and BC-3 PEL cell lines [28]. We confirmed this finding with BCBL-1 and JSC-1 lines and also assessed effects of Pom in another PEL cell line BC-2 after 2 or 3 days treatment. As seen in Fig 1A, 1B and 1C, all three cell lines showed a substantial increase in B7-2 while BCBL-1 and JSC-1 cells, but not BC-2, also showed an increase in median ICAM-1 upon Pom-treatment. To avoid possible confounding effects due to cell death, we performed our analysis using doses at which Pom led to decrease in cell proliferation but did not affect cell viability (S2 Fig). Thus, 1μM and 10μM Pom were used in BCBL-1 and BC-2 lines, and 0.2μM and 0.5μM Pom were used in JSC-1, due to differences in the sensitivity of these lines to Pom’s cytotoxic effects (S2 Fig). The lowest concentrations we tested (0.2μM Pom for JSC-1 and 1μM for BCBL-1 and JSC-1) fall around the clinically achievable cMax for Pom at 0.3μM [36].

Next, we measured the ability of the PEL cells to induce Jurkat T-cell activation in the absence or presence of Pom to determine whether these increases in B7-2 and ICAM-1 would yield improvement in T- cell activation through enhanced co-stimulatory signaling. PEL cells cultured in the absence of Pom increased the activation of Jurkat T-cells above the baseline (Fig 1D–1F). However, Pom-treated PELs further increased Jurkat T-cell activation compared to the control PEL cells in a dose-dependent manner (Fig 1D–1I). For further confirmation of T-cell activation, an ELISA for secreted IL-2 was performed to measure IL-2 produced and secreted from the endogenous IL-2 promoter in IL2-Jurkat cells. After 24 hours coincubation, Jurkat cells showed higher levels of secreted IL-2 when co-stimulated by Pom-treated PEL cells compared to control PEL cells (S1 Table). These data indicate that T-cell activation is enhanced when stimulated by PEL cells treated with Pom.

### Both ICAM-1 and B7-2 contribute to Pom-induced increase in T-cell activation

To assess the respective roles of ICAM-1 and B7-2 in the increase in Jurkat T-cell activation by Pom-treated PEL cells, we sought to block their surface ICAM-1 and/or B7-2 molecules using neutralizing monoclonal antibodies. The antibodies used for blocking were IT2.2 clone of anti-B7-2 antibody and HA58 clone of anti-ICAM-1 antibody, which have been previously shown to be inhibitory to B7-2 and ICAM-1 activity respectively [37,38]. We first validated that these antibodies do bind to their target epitopes in BCBL-1 cells. After treatment with the blocking antibodies, BCBL-1 cells were counterstained with PE-conjugated IT2.2 anti-B7-2 or HA58 anti-ICAM-1 antibodies for analysis by flow cytometry. We observed that the ICAM-1 and B7-2 blocking antibodies blocked >90% and >65% of surface ICAM-1 and B7-2 levels, respectively (S3A and S3B Fig). Further, we blocked ICAM-1 and B7-2 on the surface of an EBV-positive Burkitt’s lymphoma cell line, Daudi, which has been shown previously to enhance T-cell activation in response to increased ICAM-1 and B7-2 surface levels [29]. Blocking these molecules led to >50% reduction in T-cell activation by Daudi showing that the binding of the blocking antibodies do inhibit ICAM-1 and B7-2-mediated T-cell activation (S3C Fig).

**Fig 2:**
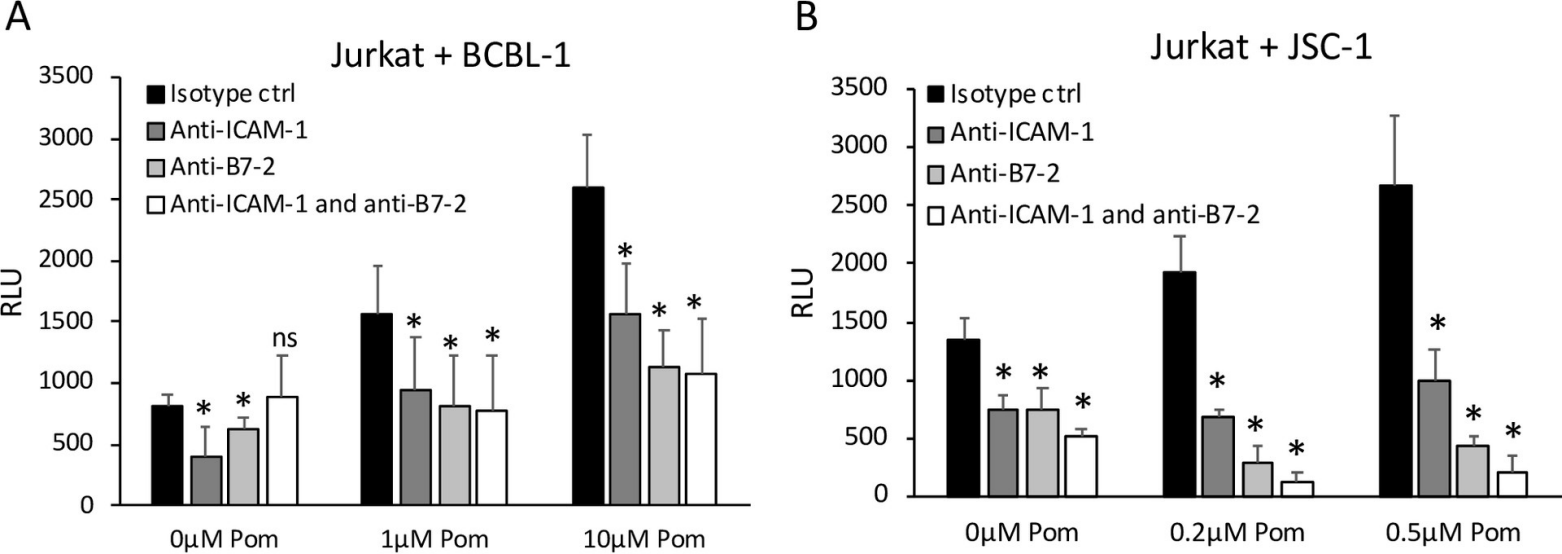
Blocking of ICAM-1 and/or B7-2 prevents Pom-induced increases in T-cell activation. PEL cells grown for 48 hours in the presence of DMSO ctrl (0μM Pom) or indicated concentrations of Pom were treated with anti-ICAM-1 Ab, anti-B7-2 Ab, both anti-ICAM-1 and anti-B7-2 antibodies, or appropriate isotype control (IgG1 for anti-ICAM-1 Ab and IgG2 for anti-B7-2 Ab), at a 10ug/mL final concentration each for 30 minutes and then co-incubated for 6 hours with IL2-Jurkat T-cells in the presence of 2.5μg/mL anti CD3 antibody. Results indicate average RLU from IL2-Jurkat cells coincubated with BCBL-1 (A) or JSC-1 (B) cells and error bars represent standard deviation from 3 independent experiments. Statistically significant differences (*p≤0.05, ns not significant paired 2-tailed *t*-test) are indicated.

Next, we blocked ICAM-1 and B7-2 on the surface of BCBL-1 and JSC-1 cells and performed Jurkat T-cell activation assays. Baseline T-cell activation by control BCBL-1 and JSC-1 cell lines could be partially inhibited by blocking either ICAM-1 or B7-2 alone (Fig 2) suggesting that the low level expression of these immune markers in the absence of Pom contribute to the baseline T-cell co-stimulation by PELs. In Pom-treated BCBL-1 cells, blocking ICAM-1 and B7-2 alone or in combination led to similar inhibitions in T-cell activation (Fig 2A), suggesting that increases in ICAM-1 and B7-2 both contribute to the Pom-induced increases in T-cell activation although increase in either is sufficient. In Pom-treated JSC-1 cells, blocking ICAM-1 or B7-2 alone significantly inhibited T-cell activation although the effect was greater when blocking B7-2, and blocking both ICAM-1 and B7-2 led to the greatest extent of inhibition (Fig 2B) suggesting that B7-2 is the primary mediator of Pom-induced increase in T-cell co-stimulation by JSC-1 cells but that ICAM-1 also plays a role. Further, unlike in BCBL-1 cells, blocking these markers in Pom-treated JSC-1 cells not only eliminated the increase caused by Pom but also reduced activation to a level below the non-Pom-treated control cells. Although B7-1 (CD80) can also enhance T-cell activation by binding to CD28, it was not expressed above the isotype controls in the PEL lines, and Pom did not increase CD80 surface expression (S4A Fig).

### Pom-treatment of both PEL and T-cells leads to greater T-cell activation than treatment of either alone

**Fig 3:**
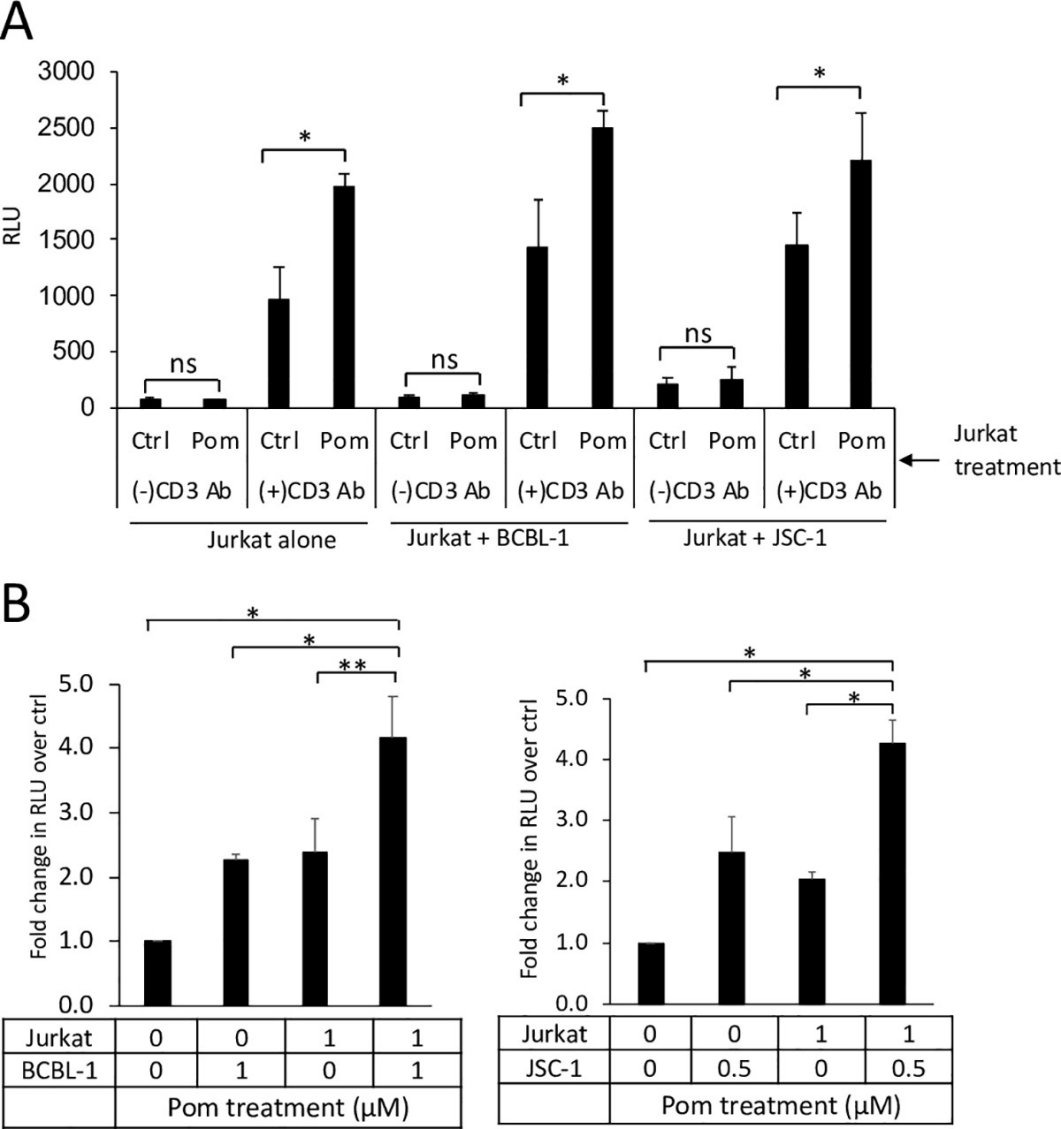
Pom treatment of both PEL and T cells induces higher Jurkat T-cell activation than treatment of either alone. (A) IL2-Jurkat cells were grown in the presence of DMSO ctrl or 1μM Pom. After 48 hours, Pom was washed out and they were incubated for 6 hours with or without control PEL cells in the presence or absence of 10μg/mL anti-CD3 antibody. Activation of ctrl or Pom-treated IL2-Jurkat cells with or without ctrl PEL cells are shown as average RLU from 3 separate experiments. (B) PEL cells and IL2-Jurkat cells were each cultured in the presence of DMSO ctrl (0μM Pom) or indicated concentrations of Pom. After 48 hours, cells were washed with PBS to remove Pom and Jurkat T-cell activation assay was performed with 10μg/mL anti-CD3 Ab. Activation of ctrl or Pom-treated Jurkat cells by ctrl or Pom-treated BCBL-1(left panel) or JSC-1(right panel) cells are shown as average fold change in RLU over non-Pom treated control from 3 independent experiments. Error bars represent standard deviation from 3 independent experiments. Statistically significant differences (*p≤0.05 and ** *p*≤0.01, ns not significant 2-tailed paired *t*-test) are indicated.

Immunomodulatory drugs, through direct activity on T-cells, can enhance PI3K activity downstream of the CD28 signaling pathway and lead to an increase in T-cell co-stimulation independent of antigen-presenting cells (APC) [19,21]. Therefore, we wanted to determine the level of T-cell activation when T-cells and PEL cells are both exposed to Pom, as would be the case *in vivo*, as compared to exposure of either alone. Pom-treated Jurkat cells showed higher activation than control Jurkat cells, not only in the absence of co-stimulation by PELs, consistent with previous reports, but also in the presence of co-stimulation by PELs (Figs 3A and S5A). We further observed that treatment of both Jurkat cells and PEL cells with Pom led to a significantly higher level of T-cell activation compared to treatment of either alone (Figs 3B and S5B). It has been reported that Pom can increase the surface expression of some immune receptors, including CD28, on primary T cells [39]. We therefore checked the surface expression levels of CD28 and CD3 in the Jurkat cells after 2 days treatment with Pom and found no change in their surface expression (S4B Fig). Overall, these data suggest that Pom-induced increases in ICAM-1 and B7-2 on PELs works in tandem with Pom’s direct activity on T-cells by increasing overall T-cell activation.

### Pom increases NK-mediated cytotoxicity of the BCBL-1 cell line

**Fig 4:**
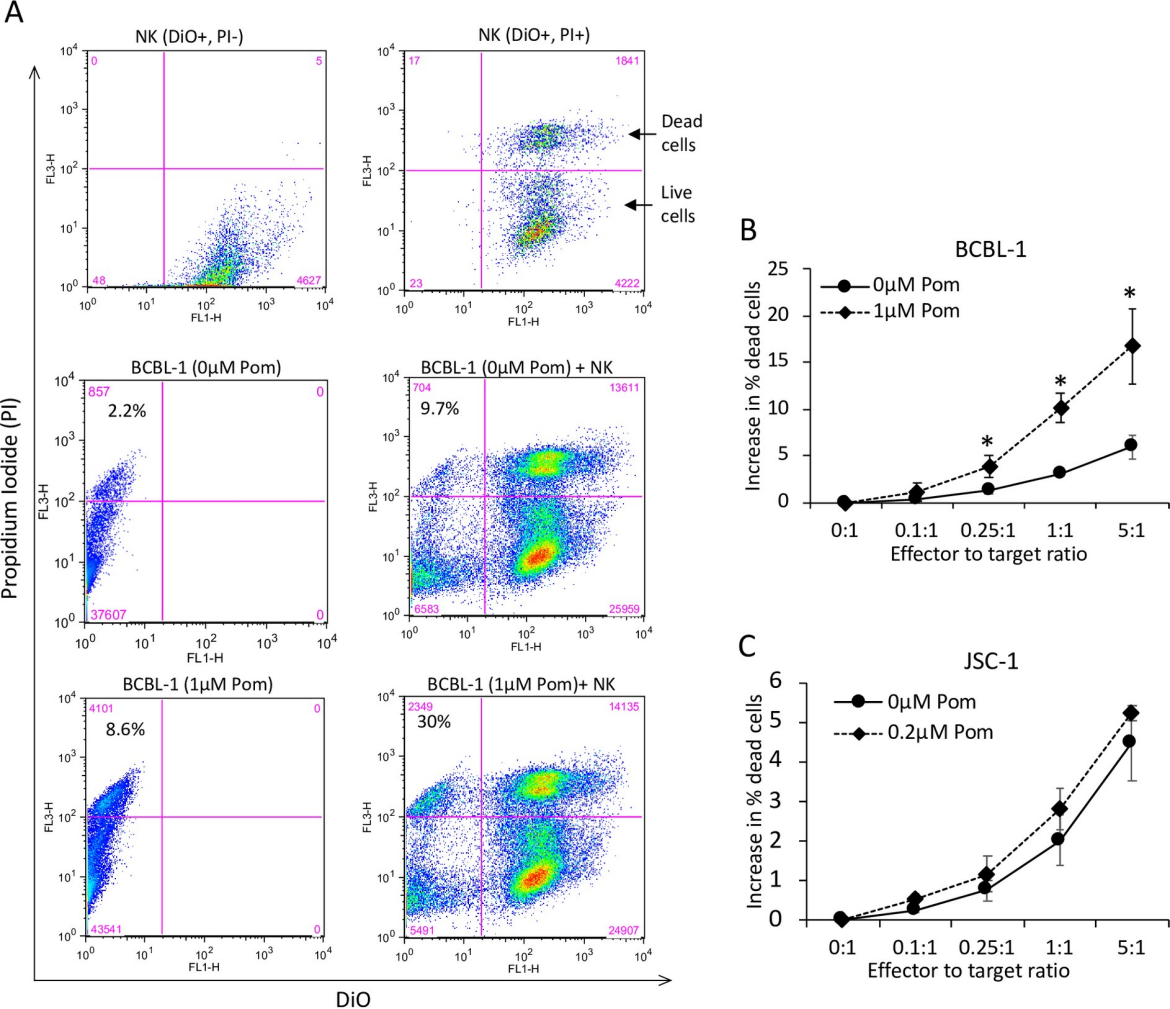
Pom increases NK-mediated cytotoxicity of BCBL-1 cells. BCBL-1 or JSC-1 cells grown for 48 hours in the presence of DMSO ctrl (0μM Pom) or indicated concentrations of Pom were coincubated with DiO-labelled YTS NK cell lines for 4 hours and then labelled with propidium iodide (PI) to determine NK-mediated cytotoxicity using flow cytometry. Note that YTS NK cells were never exposed to Pom in these experiments. (A) Representative FACS histograms showing the gating strategy. PI and DiO gates were established using DiO-positive NK cells (upper panel) with or without PI staining. Histograms for control (middle panel) or Pom-treated (lower panel) BCBL-1 cells with or without NK cells are also shown. The total number of cells within each quadrant and the percent of BCBL-1 cells that are dead (PI+ve, DiO-ve fraction) are indicated. (B and C) NK-mediated cytotoxicity of control or Pom-treated BCBL-1 (B) and JSC-1 (C) cells expressed as increase in % dead cells at various effector to target ratios. % dead cells in the absence of NK cells (0:1 effector to target ratio) was subtracted from % dead cells in the presence of NK cells to obtain NK cytotoxicity. Error bars represent standard deviation from 3 independent experiments and statistically significant differences (*p≤0.05, 2-tailed *t*-test) are indicated.

In addition to a role in facilitating T-cell activation, both ICAM-1 and B7-2 are important activating ligands for NK cells, especially those that express the CD28 receptor. Therefore, we determined if Pom could enhance NK-mediated killing of PEL. For effector NK cells, we used the YTS cell line; this line is CD28-positive and KIR-negative, so it can be activated by B7-2 but is not inhibited by MHCI [40]. To measure NK-mediated cytotoxicity, BCBL-1 cells were treated with DMSO (control) or with 1μM Pom for 2 days and then exposed to DiO-labeled NK cells at various ratios for 4 hours before measuring cytotoxicity by flow cytometry. Although exposure to NK cells led to a minor increase in death in control BCBL-1 cells (2.2% to 9.7%), it led to substantial increase in the death of Pom-treated BCBL-1 cells (8.6% to 30%) (Fig 4A). This increase was found at effector-to-target ratios ranging from 0.1:1 to 5:1 (Fig 4B). We tried repeating this experiment with JSC-1 cells; however, we were not able to perform this assay with higher than 0.2μM Pom due to higher baseline toxicity of JSC-1 cells with Pom-treatment alone. At that concentration (which had previously been shown (Fig 1B), to have a relatively small increase in ICAM-1 and B7-2), the Pom-treated JSC-1 cells did not show a significant increase in NK-mediated cytotoxicity relative to control (Fig 4C). These data suggest that Pom-treatment of PEL cells can lead to an increase in NK-mediated cytotoxicity although this effect might be variable.

### Increases in ICAM-1 and B7-2 by Pom are dependent on cereblon

**Fig 5:**
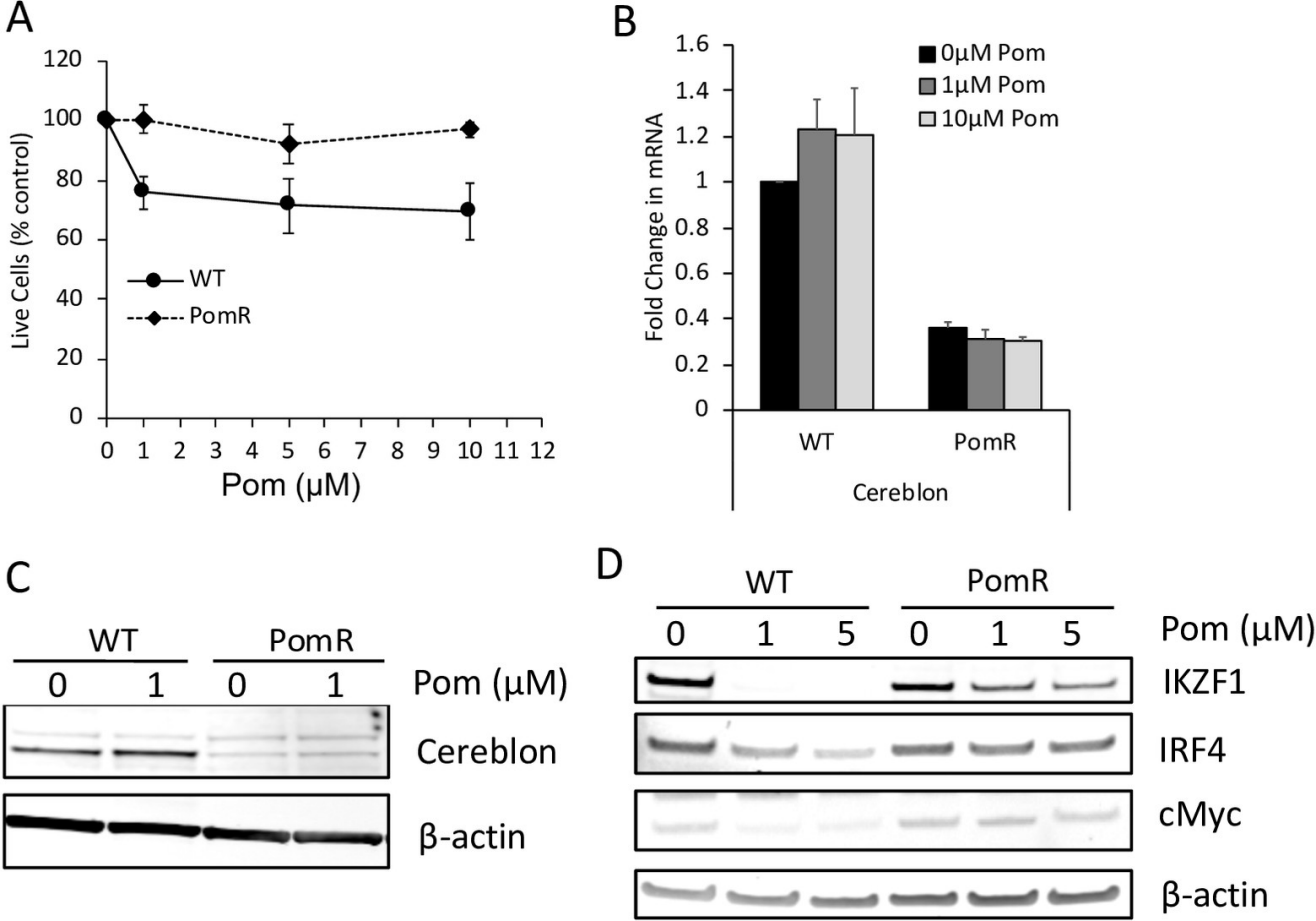
Pom-resistant PEL cells have decreased expression of cereblon. After BCBL-1 cells were passaged ~20 times with increasing concentrations of Pom in DMSO, the cells developed resistance to Pom-induced cytotoxicity (PomR cells). Control WT cells were similarly passaged but in the presence of equivalent concentration of DMSO alone. (A) Relative number of live WT or PomR cells after 48 hours treatment with DMSO control (0μM Pom) or indicated concentrations of Pom as measured by trypan blue exclusion. Shown are the mean and standard deviation from 7 separate experiments. (B) mRNA level of cereblon in WT and PomR cells in the presence DMSO control or indicated concentrations of Pom after 48 hours. mRNA levels of cereblon are normalized to that of 18S RNA and compared to WT BCBL-1 cells treated with DMSO alone (0μM Pom). Error bars represent standard error from 3 technical replicates. (C) Protein level of cereblon and β-actin control from whole cell lysates of WT and PomR cells 48 hours after Pom or control treatment. (D) Nuclear protein levels of IKZF1, IRF4, cMyc, and β-actin control in WT and PomR cells 48 hours after Pom or control treatment.

Next, we explored possible mechanisms by which Pom might increase the surface expression of ICAM-1 and B7-2 in PELs. Most of the immunomodulatory activities of CBIs in immune effector cells as well as in tumor cells are dependent on their binding to cereblon and the resulting downstream changes. In multiple myeloma cells, CBIs raise the levels of the NK-activating ligands MICA and PVR through cereblon-dependent degradation of IKZF-1/3 and IRF4 [26]. Cereblon loss is a common mechanism by which multiple myeloma cells develop resistance to cytotoxic activity of Pom and other CBIs [17,41]. Therefore, we wanted to first determine whether PELs can develop resistance to Pom’s toxicity after *in vitro* exposure with increasing concentrations and if so, whether the resistance is associated with a loss of cereblon expression. After culturing BCBL-1 cells for approximately 20 passages with increasing concentrations of Pom, their growth was no longer affected by up to 10μM Pom (Fig 5A). These Pom-resistant cells (PomR) had a 70% decrease in cereblon mRNA levels and a nearly 90% decrease in the protein levels (Fig 5B and 5C). While the levels of IKZF1, IRF4, and cMyc were similar in untreated PomR and WT cells, treatment with either 1μM or 10μM Pom only weakly affected the expression of IKZF-1, IRF4, and cMyc in PomR cells, whereas Pom eliminated the detectable IKZF-1 and significantly decreased the levels of IRF4 and cMyc in WT cells (Fig 5D). This result could be explained by the decreased expression of cereblon in PomR cells (Fig 5C). These observations indicate that, similar to multiple myeloma cells, PELs can develop resistance to immunomodulatory drugs through downregulation of cereblon.

**Fig 6:**
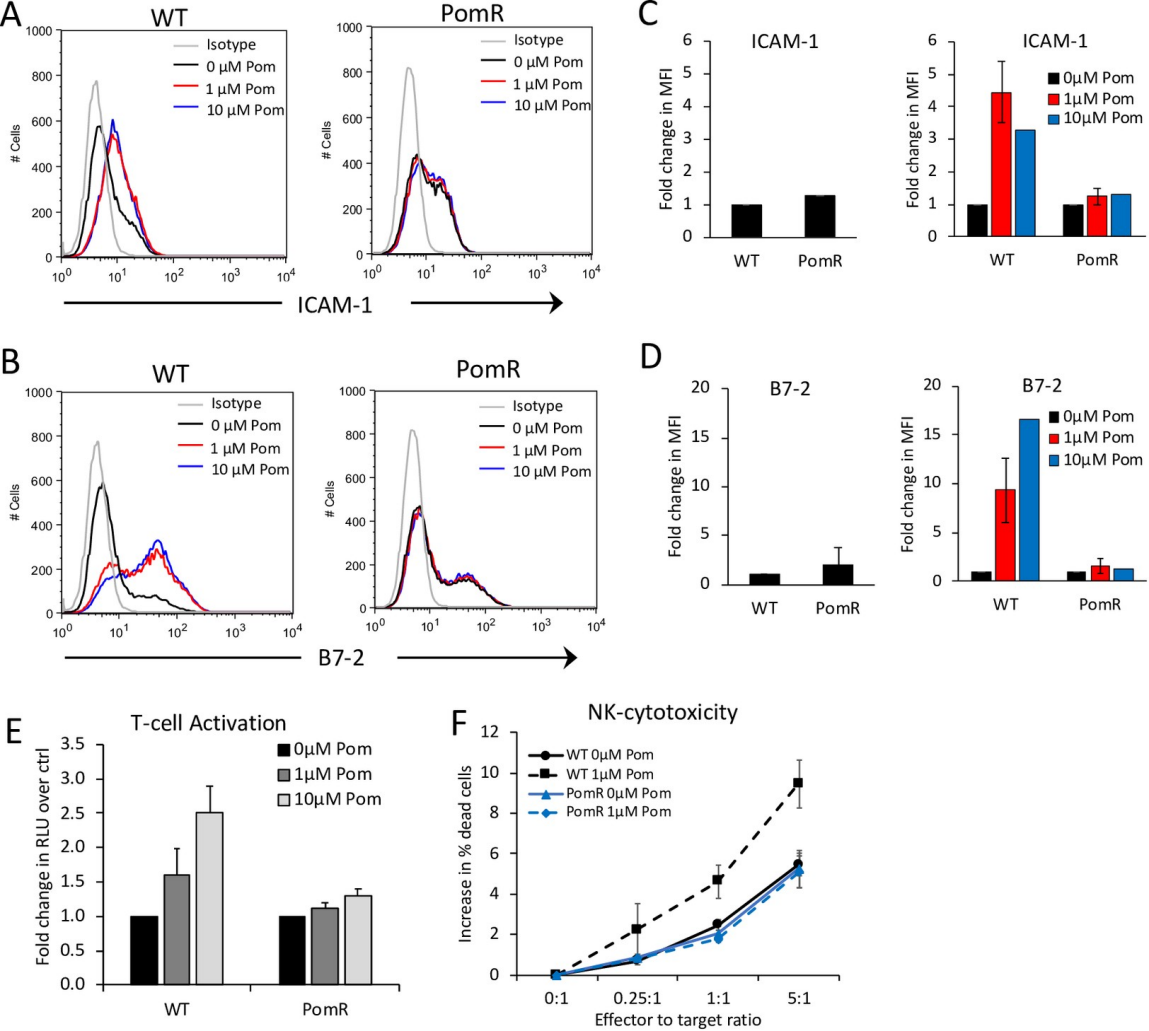
Pom does not increase ICAM-1 and B7-2 surface expression or T-cell and NK-cell recognition of Pom-resistant cells. WT and PomR BCBL-1 cells were cultured in the presence of DMSO ctrl (0μM Pom) or indicated concentrations of Pom. After 48 hours, surface expression levels of ICAM-1 and B7-2 were measured as well as Jurkat T-cell and YTS NK-cell activation by the WT and PomR cells were determined. (A and B) ICAM-1 (A) and B7-2 (B) levels determined using flow cytometry after staining with FITC-conjugated IgG isotype control, anti-ICAM-1, or anti-B7-2 antibodies. (C and D) Mean fold changes in median fluorescence intensity (MFI) of ICAM-1 (C) and B7-2 (D) from 4 and 6 independent experiments, respectively (except 10μM Pom-treatment, which was done once). Left panels show fold changes in ICAM-1 and B7-2 in PomR BCBL-1 cells relative to WT BCBL-1 cells. Right panels show fold changes in ICAM-1 and B7-2 in Pom-treated WT and PomR BCBL-1 cells relative to DMSO ctrl-treated cells. (E) T-cell activation of IL2-Jurkat cells by WT or PomR BCBL-1 cells after 6 hours co-culture in the presence of 10μg/mL anti CD3 antibody. Activation is expressed as average fold change in luciferase activity by Pom-treated cells over DMSO ctrl-treated cells from 3 independent experiments. (F) NK-mediated cytotoxicity of WT or PomR BCBL-1 cells after 4 hours co-culture with YTS NK cell line. NK-cytotoxicity was measured by flow cytometry and expressed as average increase in % dead cells in the presence of NK cells at various effector to target ratios from 3 independent experiments. Error bars represent standard deviations.

**Fig 7:**
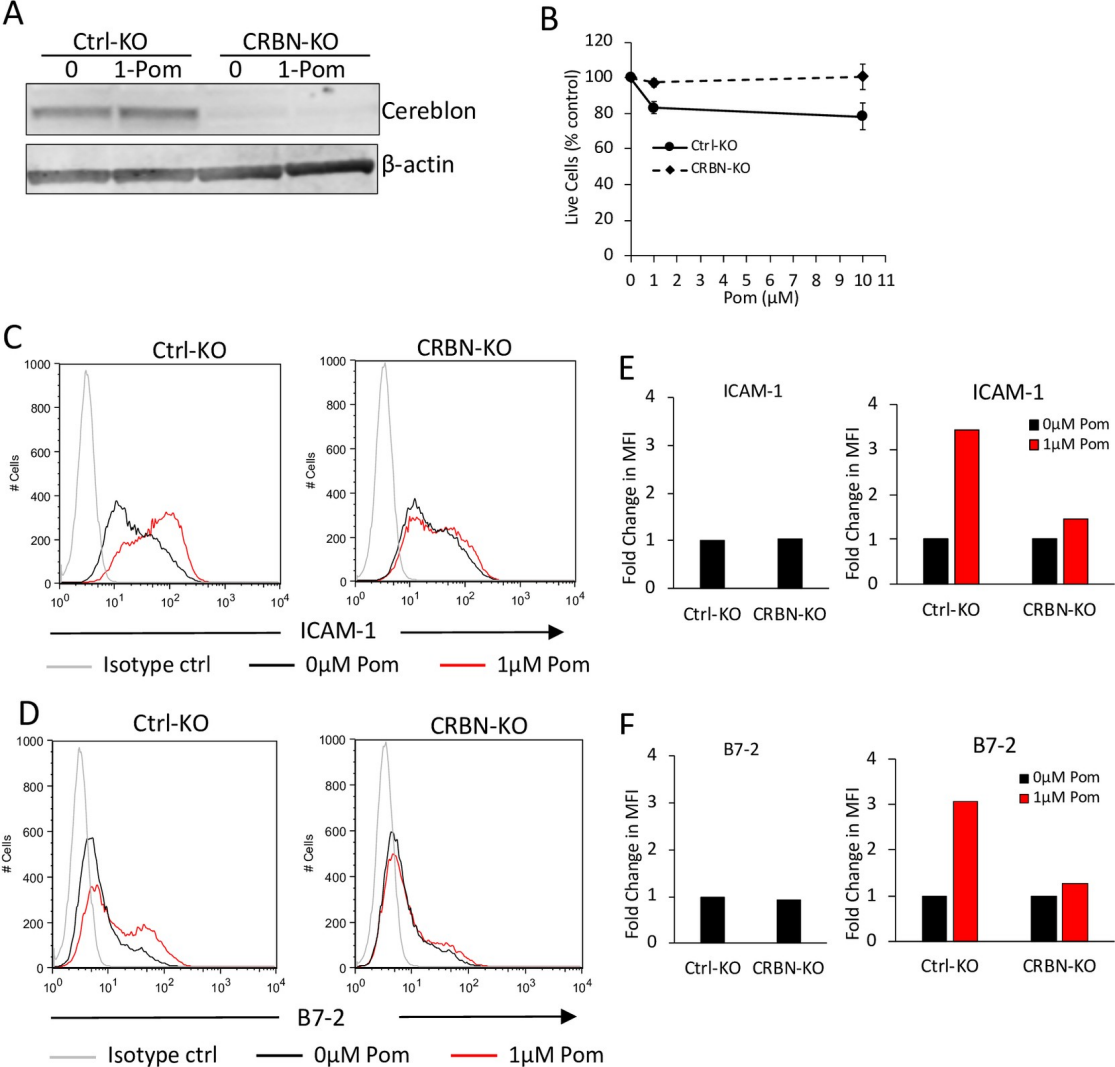
Cereblon is necessary for Pom-induced increases in ICAM-1 and B7-2 surface expression. (A) Protein levels of cereblon (CRBN) and β-actin control from whole cell lysates of Ctrl-KO or CRBN-KO cells 48 hours after Pom or DMSO-ctrl (0μM Pom) treatment. (B) Relative number of live Ctrl-KO or CRBN-KO cells after 48 hours treatment with indicated concentrations of Pom as measured by the trypan blue exclusion method from 3 separate experiments. (C and D) ICAM-1 (C) and B7-2 (D) levels determined using flow cytometry after staining with PerCP/Cy5.5-conjugated IgG isotype control, anti-ICAM-1, or anti-B7-2 antibodies. (E and F) Fold changes in median fluorescence intensity (MFI) of ICAM-1 (E) and B7-2 (F) from histograms shown in (C) and (D) respectively. Left panels show fold changes in ICAM-1 and B7-2 in CRBN-KO BCBL-1 cells relative to Ctrl-KO BCBL-1 cells. Right panels show fold changes in ICAM-1 and B7-2 in Pom-treated Ctrl-KO or CRBN-KO cells relative to DMSO-ctrl treated cells. This experiment was performed 3 times with similar results; one representative experiment is shown.

We next assessed the effects of Pom on ICAM-1 and B7-2 on the PomR cells with decreased cereblon. In the absence of Pom, PomR cells didn’t express significantly different levels of ICAM-1 or B7-2 relative to WT cells (Fig 6A–6D). However, while 48 hours of treatment with 1μM or 10μM Pom led to substantial increases in ICAM-1 and B7-2 in WT cells, no such increases were observed in PomR cells (Fig 6A–6D). Also, Pom treatment of PomR BCBL-1 cells failed to increase T-cell activation (Fig 6E) or NK-mediated killing (Fig 6F). To directly test the role of cereblon, we next utilized a cereblon knock-out (CRBN-KO) BCBL-1 cell line generated using CRISPR/Cas9 methodology (Fig 7A). CRBN-KO cells, similar to PomR cells, showed resistance to Pom-induced toxicity (Fig 7B). Further, CRBN-KO cells expressed similar levels of basal ICAM-1 and B7-2 levels compared to control knock-out BCBL-1 cells (ctrl-KO cells) and only showed a minor increase in ICAM-1 and B7-2 upon Pom-treatment compared to ctrl-KO cells, which showed substantial increases in these markers (Fig 7C–7F). These observations together provide evidence that the increase in the levels of ICAM-1 and B7-2 on PELs and the resulting increase in T-cell and NK-cell activity are dependent on Pom’s ability to modulate cereblon activity.

We further aimed to determine if the decreases in the levels of IKZF1, IRF4, or cMyc caused by Pom-cereblon interactions are responsible for the increases in ICAM-1 and/or B7-2. JQ-1, a small molecule inhibitor of bromodomain-containing 4 (BRD4), has been shown to inhibit the expression of cMyc and IRF4 [42]. It can also suppress IKZF1 expression in multiple myeloma cells [43]. Based on these findings, we used JQ-1 to bypass cereblon and directly suppress the levels of IKZF1, IRF4, and cMyc in PELs. JQ-1 suppressed the growth of BCBL-1 cells (S6A Fig) as well as the levels of IKZF1, IRF4, and cMyc in a dose-dependent manner (S6B Fig) in these cells. Despite decreases in these factors, JQ-1 treatment did not show dose-dependent increases in ICAM-1 or B7-2 (S6C Fig) providing evidence that decreases in these downstream targets of Pom-cereblon are not in themselves sufficient for Pom-induced increases in the surface expression of ICAM-1 and B7-2. It should be noted, however, that although JQ-1 lowered the levels of IKZF1 it was not able to eliminate its detection as is observed with Pom-treatment (see Fig 5).

### Pom-induced increases in ICAM-1 and B7-2 requires PI3K pathway

We next explored some of the potential mechanisms that might be involved in the modulation of ICAM-1 and/or B7-2 in APCs. In some macrophages and B-cells, IL-10 can decrease levels of surface B7-2 by increasing MARCH 1 and 8, which can ubiquitinate and downregulate B7-2 on the cell surface [44,45]. CD83 can block MARCH protein-induced degradation of B7-2 [46]. We hypothesized that Pom might alter this pathway to induce an upregulation of B7-2 and ICAM-1. We found that Pom decreases IL-10 in BCBL-1 but increases it in JSC-1 cells (S7A Fig). Moreover, Pom did not change the levels of MARCH 1 or MARCH 8 mRNAs, nor did it change the level of CD83 at the surface (S7B and S7C Fig). Furthermore, neither blocking IL-10 signaling using anti-IL-10 antibody nor supplementing the PEL cells with exogenous recombinant human IL-10 (rIL-10) led to changes in ICAM-1 and B7-2 surface expression (S7D and S7E Fig). Taken together, these results suggest that Pom-induced increases in these markers do not involve alterations in the IL-10/MARCH pathway.

**Fig 8:**
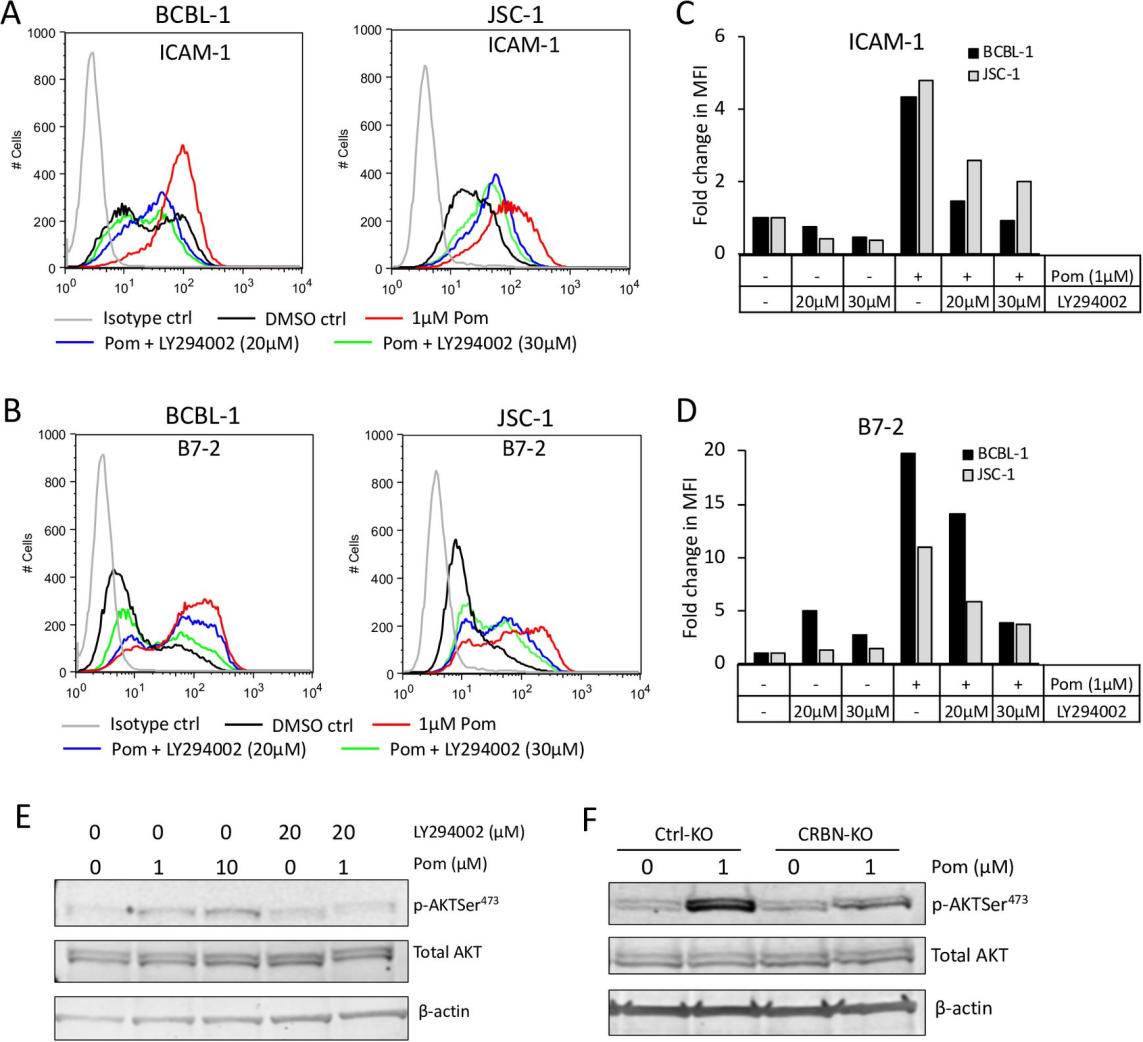
Activation of the PI3K pathway by Pom plays a role in the increases in ICAM-1 and B7-2. PEL cells were pretreated with PI3K inhibitor LY294002 for approximately 1 hour and then treated with Pom for an additional 48 hours. (A and B) Surface expression levels of ICAM-1 (A) and B7-2 (B) measured by flow cytometry using PerCP/Cy5.5-conjugated anti-ICAM-1 or anti-B7-2 antibodies in BCBL-1 and JSC-1 cells treated with Pom (1μM) and/or LY294002 (20 or 30 μM). (C and D) Fold changes in median fluorescence intensity (MFI) of ICAM-1 (C) and B7-2 (D) upon treatment with Pom and/or LY294002 relative to DMSO-treated control cells. (E) Western blot analyses of cytoplasmic extracts of BCBL-1 cells plated with Pom and/or LY294002 for Phospho and total AKT. (F) Western blot analysis of whole cell lysates of Ctrl-KO or CRBN-KO BCBL-1 cells treated for 48 hours with DMSO control or 1μM Pom. β-actin is used as a loading control. These experiments were performed 3 times and the results from one representative experiment are shown.

STAT3 and PI3K pathways are also involved in regulating the expression of various immune markers, including ICAM-1 and B7-2, in response to various stimuli such as IL-21 and B cell receptor (BCR) engagement [47–49]. We first explored the role of STAT3. However, the STAT3 inhibitor S3I-201 did not alter ICAM-1 or B7-2 levels on its own (S8A Fig) nor did it prevent their upregulation by Pom (S8B Fig). Previous studies have shown that IKZF1 can induce the expression of miR-26b, and this in turn leads to inhibition of PI3K pathway [50]. We thus hypothesized that Pom, by eliminating IKZF1, might activate the PI3K pathway, leading to increased surface expression of ICAM-1 and B7-2. To test this hypothesis, we treated PEL cell lines with LY294002 (a PI3K inhibitor) with or without Pom. LY294002 dose-dependently abrogated Pom-induced increases in both ICAM-1 and B7-2 (Fig 8A–8D) although it induced only minor and inconsistent changes in these markers when used alone (Fig 8C and 8D). Further, we measured if PI3K activity is increased upon Pom-treatment by measuring the level of phosphorylated AKT (pAKTSer^473^), a vital downstream mediator of PI3K signaling. Pom-treatment led to an increase in pAKTSer^473^ in BCBL-1 cells, and this increase was prevented by LY294002 (Fig 8E). Additionally, ctrl-KO BCBL-1 cells showed a larger increase in pAKTSer^473^ compared to CRBN-KO BCBL-1 cells even though the two lines had similar baseline pAKTSer^473^ level (Fig 8F). Together, these data provide evidence that Pom-induced increase in PI3K activity plays an important role in the increases in ICAM-1 and B7-2.

## Discussion

We previously demonstrated that the thalidomide derivatives Len and Pom could increase immune surface marker expression in KSHV-infected PEL cells [28]. Here, we show that treatment of PEL cells with Pom restores NK cell lysis and T-cell activation, and that this is directly due to the upregulation of the co-stimulatory molecules ICAM-1 and B7-2, as specific blocking antibodies to these proteins prevent the responses. To our knowledge, this is the first report of otherwise “immunologically silent” PELs being specifically sensitized for recognition and lysis by T-cells and NK-cells, both of which are important components of anti-tumor immunity. Our mechanistic studies further provide evidence that these effects occur through Pom’s effects on the E3 ubiquitin ligase cereblon and, at least in part, involve the PI3K pathway.

Most oncogenic viruses have evolved mechanisms to inhibit various components of the antigen-presentation pathway to suppress recognition of the virus-specific antigens. KSHV in particular decreases co-stimulatory molecules ICAM-1 and B7-2, decreases MHCI and MHCII surface expression (especially upon lytic activation), impairs intracellular antigen processing, and decreases TAP-1-mediated MHCI transport; thus KSHV infection in general results in poor T-cell-mediated immune responses [4–9]. Hence, immunotherapy modalities that restore antigen-presentation pathways and enhance specific T-cell responses hold promise for the treatment of KSHV-associated tumors, including PEL. While some KSHV proteins, particularly lytic proteins, do elicit T-cell responses, none of the antigens are immunodominant, and the T-cell response is weak compared to that elicited by other viruses [51,52]. Among KSHV-infected individuals, the risk of KS increases substantially in the face of immunosuppression due to HIV-AIDS or during post-transplantation. In patients with AIDS-KS, KS often regresses after reconstitution of immunity by combination antiretroviral therapy (cART), which is associated with an increase in KSHV-specific T-cell responses [51,53].

*In vitro* studies show that PEL cell lines elicit a poor T-cell response compared to cell lines derived from some other lymphoid malignancies ([30], S1 Fig). The exact reason for these findings is incompletely understood, but they are consistent with the general observation that PEL cell lines express lower levels of MHCI as well as ICAM-1 and B7-2 on their surface ([30], S1 Fig). In addition to these positive signaling molecules, T-cell responses can be modulated by interactions between negative signaling molecules on antigen-presenting cells/tumor cells and their receptors on T-cells. In one study done to compare the expression level of PD-L1, one of the important negative immunomodulatory molecules, in several primary tumors, PELs were found to be weekly positive while EBV-positive Burkitt’s lymphoma were found to be negative for PD-L1 [54]. Thus, it is possible that the higher PD-L1 levels in PELs or potential differences in the levels of other negative signaling molecules on their cell surface, at least partially, contribute to these observed differences in T-cell responses induced by these different types of cancers. This said, there is evidence that T-cell immune responses can play an important role in the control of this tumor. While nearly all PELs develop in the setting of AIDS, patients often have more than 100 CD4^+^ cells/μL [1]. Also, in a recent study in patients with HIV and advanced cancer, one of 2 patients with PEL responded to pembrolizumab, an anti-PD-1 drug [55]. Thus, PEL can be responsive to T-cell control, and strategies that enhance T-cell immunity to this tumor are worth exploring.

In this report, we show that Pom enhances the recognition of PELs by the Jurkat T-cell line through increased co-stimulatory signaling provided by increased expression of ICAM-1 and B7-2 on the PEL cells. The dose of Pom required to increase T-cell activation as well as the degree to which each of the two markers contribute to Pom-induced increases in T-cell responses varied among the cell lines. The exact reason for these differences are not known but could in part be due to the variable presence of EBV and differences in the degree of lytic activation of KSHV. Additionally, these lines might have variation in the levels of other positive as well as negative immunomodulatory signaling molecules on their surface that could further contribute to this observation. During lytic reactivation, KSHV-infected cells suppress surface MHCI levels as one of the strategies to prevent presentation of KSHV-derived antigens and recognition of reactivating cells by the hosts’ immune surveillance machinery, particularly T-cells (reviewed in [3]). We previously found that Pom prevents downregulation of MHCI during lytic reactivation of PEL cell lines [28]. However, due to limitations in obtaining HLA-matched T-cell clones specific to the KSHV antigens in the PEL cell lines, we could not assess the additional effects of increased MHCI in this report. However, since MHCI is involved in antigen-presentation to T-cells, Pom-induced increase in MHCI, particularly during lytic replication, would be expected to further increase T-cell activation in the context of MHCI-mediated T-cell stimulation, and thus aid in the removal of lytically-activated KSHV-infected cells to prevent or reduce further production of KSHV virions. That said, there is evidence, based on murine models, that an increase in B7-2 alone can lead to a functional increase in T-cell activity *in vivo* [30].

In addition to T-cell immunity, NK-cell-mediated innate immunity also plays an important role in controlling KSHV-associated diseases. Clearance of KSHV and regression of KS with cART in patients with advanced AIDS-KS are both associated with a restoration of NK-immunity, and failure to show a clinical response with cART is associated with impaired NK-immunity [56]. Further, NK cells derived from advanced AIDS-KS patients, but not healthy individuals, are deficient in their ability to target cells latently infected with KSHV, suggesting that the progression of KSHV-associated diseases is associated with the expansion of NK-cell population that respond poorly to KSHV-infected cells [56]. Therefore, increasing NK-mediated cytotoxicity is likely to aid in the treatment of PELs. Here, we demonstrated that Pom-treated PEL cells can be made more susceptible to NK-cell mediated cytotoxicity as measured using an NK-cell line YTS. However, we only observed significantly increased NK-mediated killing of Pom-treated BCBL-1 but not JSC-1. As noted above, Pom also increases expression of MHCI on those cells undergoing lytic KSHV activation [28], and this can be inhibitory to NK-cells. Thus, further work needs to be done to clarify whether Pom-treatment would result in increased NK-mediated killing of PEL cells *in vivo* although our data provides evidence that this would occur, especially in the majority of PEL cells that are not undergoing lytic KSHV activation.

Most of Pom’s effects are mediated by Pom’s interaction with cereblon, a cellular E3 ubiquitin ligase. Here, we found evidence that the increases in ICAM-1 and B7-2 by Pom are also dependent on cereblon. First, we found that PEL cells exposed to increasing concentratins of Pom can develop resistance to its cytotoxic effects, and that this resistance is associated with a loss of cereblon expression, similar to findings in MM cells [17,41]. This data is also consistent with a previous report by Patil et al. in which cereblon was identified using targeted genetic approach to be one of the two genes whose inactivation leads to Pom-resistance in PEL cell lines [57]. Our data shows that while short-term Pom-treatment does not significantly alter cereblon levels in PEL cells, long-term Pom-treatment can decrease its levels. The exact mechanism by which PEL cells have decreased cereblon expression after long-term Pom exposure is not completely understood. Unlike in MM cells, cereblon does not contribute to the growth or viability of PEL cells [18,24,41], suggesting that the mechanism may differ somewhat from that in MM. Furthur work needs to be done to understand the mechanism for this effect.

The BCBL-1 line that developed resistance to Pom *in vitro* had a muted response to Pom treatment with diminished downstream effects ascribed to cereblon. Both the Pom-resistant as well as CRBN-KO BCBL-1 cell lines failed to show significant increases in the immune surface markers upon Pom-treatment, providing strong evidence that the effects of Pom on surface immune markers were mediated through cereblon. We then explored the role of the known downstream targets of Pom-cereblon interaction, IRF4 and cMyc. While we have previously demonstrated an inverse correlation between the levels of IRF4 and the upregulation of immune surface markers in the presence of Pom [28], we were not able to show that downregulating IRF4 expression was in itself sufficient to upregulate the markers. The BET bromodomain inhibitor JQ-1 was able to decrease IRF4 and cMyc levels but this did not lead to increases in ICAM-1 and B7-2, suggesting that either IRF4 and cMyc have no role or that other factors may also be required for this effect. Another possibility is that other effects of JQ-1 could be masking the effects of IRF4 and cMyc downregulation on ICAM-1 and B7-2 regulation.

We also investigated other pathways known to be involved in the regulation of these molecules, and found that Pom does not interfere with the IL-10/MARCH or STAT3 pathway to upregulate the markers. However, our data strongly suggests that Pom uses the PI3K pathway at least in part to upregulate both ICAM-1 and B7-2. While we did not investigate the mechanism by which Pom alters the PI3K pathway in PELs, it likely involves IKZF1, which has been shown to suppress PI3K pathway in T-cell acute lymphoblastic leukemia cells [50]. Here, we find that Pom activates PI3K pathway in BCBL-1 PEL cell line as shown by an increase in phosphorylated AKT, consistent with a previous report where Pom was shown to activate this pathway in BL cells that are infected with a closely related herpesvirus, EBV [58]. It is likely that the effects of PI3K on surface immune markers are mediated at least in part through mTOR, an important downstream mediator of PI3K signaling. In MM cells, Pom has been shown to increase mTOR activity [59], which, in some dendritic cells, has been shown to be necessary for the expression of co-stimulatory molecules including ICAM-1 and B7-2 [49]. Since the mTOR signaling pathway is key in regulating mRNA translation, and our previous study suggests that Pom-induced increases in ICAM-1 and B7-2 does not involve increases in transcription [28], we propose a model where Pom-induced degradation of IKZF-1 leads to an increase in PI3K/mTOR activity which then leads to increased translation of the immune surface markers in PELs. It should be noted, however, that PI3K inhibition did not completely abrogate Pom-induced increases in ICAM-1 and B7-2; therefore, it is likely that the increases in these markers involve additional mechanisms. One possibility is that Pom could interfere with KSHV-mediated degradation of ICAM-1 and B7-2, and at least partially contribute to increases in ICAM-1 and B7-2. The levels of K3 and K5, two lytic proteins of KSHV known to ubiquitinate and degrade these markers at the cell surface, were either unchanged or slightly upregulated upon Pom-treatment of PEL cell lines during latency [28]. However, we have previously found that their expression is inhibited by Pom in lytically reactivated PEL cells [28] suggesting that the changes in K3 and K5 levels could play a role during lytic reactivation but are not likely to be responsible for the Pom-induced increases in the ICAM-1 and B7-2 levels in PEL cells not activated to lytic KSHV replication. This said, we cannot rule out the possibility that Pom can reduce K3 and K5 activity even during latency by altering other components of the K3/K5-associated ubiquitination complex such as UbcH5 and Ubc13 (reviewed in [8]).

In summary, we show here that Pom can specifically activate immune response against KSHV-infected tumor cells by restoring immune molecules required for effective NK and cytotoxic-T cell recognition on the surface of PEL cells. Unlike the other immune effects of Pom that involve induction of general immune stimulation through activity on immune effector cells [19–23], this activity works at the target cell level and thwarts virus-induced mechanisms that normally function to evade immune recognition and thus makes the virus-infected tumor cells more susceptible to immunologic killing. The fact that Pom can both activate the effector cells and at the same time increase recognition of the target tumor cells strongly supports the study of Pom, alone or in combination with other therapies, in the treatment of PEL and other KSHV-associated diseases.

## Materials and methods

### Cell culture reagents

Pom was obtained from Celgene Corp. (now Bristol Myers Squibb) and was stored as frozen stocks (20–100 mM) in dimethyl sulfoxide (DMSO)(Sigma). JQ-1, LY294002, and S3I-201 were purchased from Selleck Chemical (Houston, TX) and stored in DMSO. Anti-human IL-10 antibody (cat# AF-217-NA) and recombinant human IL-10 protein (rIL-10) (cat# 217-IL-010) were purchased from R&D systems (Minneapolis, MN).

### Cell culture

BCBL-1, JSC-1, BC-3, Raji, Daudi, and YTS cells were obtained and maintained as described previously [28,29]. MC116 and BC-2 cells were obtained from ATCC (Manassas, VA). The IL2-Jurkat T cell line was obtained from Promega (Madison, WI, cat # J1651) and grown in complete media with 200μg/mL hygromycin. Except for experiments involving resistant cells, all the cell lines were grown in culture for a maximum of 20 passages after thawing. Cell lines were also tested for mycoplasma at various points and found to be negative. Pom-resistant (PomR) BCBL-1 cells were obtained by culturing BCBL-1 cells for approximately 20 passages with increasing concentrations (0.5μM to 10μM) of Pom. As a control, BCBL-1 cells were cultured for the same number of passages without Pom but with an equivalent concentration of DMSO to generate the matched wild-type (WT) BCBL-1 cells. Stocks of PomR and matched WT BCBL-1 cells were frozen down early after the generation of PomR cells. For use in experiments, the PomR and WT cells were thawed from frozen stocks and maintained in culture in the absence of Pom/DMSO (unless otherwise noted) for a maximum of approximately 1 month. No reversal of resistant phenotype was observed in PomR cells within this duration. BCBL-1 cereblon-knockout (CRBN-KO) and control-knockout (ctrl-KO) cells were a kind gift from Dr Eva Gottwein from Northwestern University, Evanston IL [57].

### Real-time quantitative PCR (RT-qPCR)

RT-qPCR was performed as described previously [28]. mRNA expression was normalized to that of 18S endogenous control RNA and the quantification of relative mRNA expression was performed using ΔΔCt method. Primers for 18S (GCCCGAAGCGTTTACTTTGA and TCCATTATTCCTAGCTGCGGTATC) were synthesized by Lofstrand Labs Limited (Gaithersburg, MD). Primers for cereblon (qHsaCED0001537), MARCH 1 (qHsaCID0010480), and MARCH 8 (qHsaCID0007297) were purchased from Bio-Rad (Hercules, CA) as PrimePCR SYBR Green q-PCR assays (cat# 10025636).

### Flow cytometry for surface expression

Analysis of surface marker expression was carried out by flow cytometry as described previously [28] using FITC or PerCP/Cy5.5-conjugated antibodies. FITC-conjugated antibodies used were anti-CD3 (BD Biosciences, cat # 349201), anti-CD28 (BD Biosciences, cat # 561790), anti-CD83 (BD Biosciences, cat# 556910), anti-CD86 (Abcam, Cambridge, MA, cat # Ab77276), anti-CD54 (Santa Cruz, Dallas, TX, cat # SC-107), and IgG2a isotype control (Sigma, cat # F6522). PerCP-Cy5.5-conjugated antibodies anti-CD86 (cat # 374215), anti-CD54 (cat # 353119), anti-CD80 (cat # 305231), and IgG1κ isotype control (cat # 400150) were purchased from Biolegend, San Diego, CA.

### Western blot analysis

Cells were plated at 3 x 10^5^ cells per mL for 48 hours in the absence or presence of Pom. Whole cell lysates or nuclear and cytoplasmic extracts were prepared using M-PER mammalian protein extraction reagent or NE-PER Nuclear Extraction kit, respectively (ThermoFisher Scientific, Waltham, MA) according to the manufacturer’s protocol. Lysates used for the analyses of phosphorylated AKT were extracted in the presence of both phosphatase inhibitor cocktail and protease inhibitor cocktail (ThermoFisher Scientific) at a final 1X concentration. Protein concentrations were determined using BCA Protein Assay Kit (ThermoFisher Scientific). Equal amounts of protein were run on 4 to 12% NuPAGE Bis-Tris precast gels (ThermoFisher Scientific) and western blot analyses were performed and analyzed using the Odyssey imaging system and ImageStudio software (Li-Cor) as described previously [28]. The primary antibodies used were mouse anti-β-actin (Sigma, cat# A2228), mouse anti-IKZF1 (Santa Cruz, cat# 398265), rabbit anti-IRF4 (Cell Signaling, Danvers, MA, cat# 4964), and rabbit anti-cMyc (Abcam, cat# Ab11917). Primary antibodies against cereblon were rabbit anti-cereblon Ab from two sources, cat# HPA045910 from Sigma and CRBN65 Ab [60] kindly provided by Celgene Corp. Primary antibodies against AKT were rabbit anti-phospho (Ser473)-AKT (cat# 4060) and mouse anti-AKT-pan (cat# 2920) from Cell Signaling.

### T-cell activation assays

T-cell activation assays were performed using the T-cell activation bioassay kit (Promega, cat# J1651) according to the manufacturer’s recommended protocol. Briefly, PEL cells (3x10^5^ cells per mL) were treated with the indicated concentrations of Pom for 2 or 3 days, after which cells were washed with PBS to remove Pom and then T-cell activation was assessed using IL2-Jurkat cells (Jurkat T-cells expressing a luciferase reporter gene under IL-2 promoter) as the effector T cells. IL2-Jurkat T-cells (10^5^ cells) were stimulated using various concentrations of anti-human CD3 monoclonal antibody (OKT3 from ThermoFisher Scientific, cat# 16-0037-81) and co-stimulated with 2x10^5^ control or Pom-treated PEL cells at a 2:1 ratio (PEL to Jurkat) in a 37°C incubator. The assay was carried out in triplicate in 96 well-plates containing 25μL of PEL cells, 25μL of Jurkat cells, and 25μL of anti-CD3 antibody per well and incubated for 6 hours at 37°C before adding bio-glo reagent (Promega). Relative light units (RLU) were measured using Victor X3 multilabel plate reader (PerkinElmer). Background luminescence from wells without cells was subtracted from all the wells containing cells. Luminescence data was plotted as a 4PL regression graph using GraphPad Prism software. Fold change in activation by Pom was determined after subtracting baseline RLU obtained from Jurkat cells without co-stimulation by PELs from that obtained with co-stimulation by PELs. T-cell activation assays of non-PEL cell lines were performed similarly except at a 1:5 target to effector ratios.

### Blocking experiments

PEL cells grown in the presence or absence of Pom for 2 days were resuspended at 16x10^6^ per mL in T-cell activation assay buffer. Blocking antibodies (ThermoFisher Scientific) used were CD86 (B7-2) monoclonal antibody clone IT2.2 (16-0869-81) and CD54 (ICAM-1) monoclonal antibody clone HA58 (16-0549-82). Isotype-matched control antibodies used were IgG2bκ monoclonal antibody clone eBMG2b (16-4732-82) for CD86 and IgG1κ monoclonal antibody clone P3.6.2.8.1 for CD54 (16-4714-82). Blocking reactions were carried out in a 96-well plate and each reaction contained 100μL PEL cells, 40μL each of two antibodies (test and/or isotype-matched control antibody to a final 10μg/mL concentration) and 20μL buffer. The reaction was incubated for 30 minutes at room temperature and either used directly in T-cell assay (25μL per well) in triplicate as described above or counter-stained with PE-conjugated anti-ICAM-1 (clone HA58, cat# 12-0549-42) or anti-B7-2 (clone IT2.2, cat# 12-0869-42) antibodies from Thermofisher Scientific to assess the surface levels of ICAM-1 and B7-2 by flow cytometry for validation of binding and blocking activities of the blocking antibodies. Daudi cells were blocked with the blocking antibodies similarly and then directly used in T-cell assay.

### ELISA

To perform ELISA for IL-2, PEL cells were cultured at 3x10^5^ cells per mL in the absence or presence of Pom for 48 hours. 4x10^5^ PEL cells were then co-cultured with 2x10^5^ Jurkat cells in the presence of 2.5μg/mL anti-CD3 antibody for 24 hours. The assay was carried out in triplicate in a 96 well-plate containing 50μL PEL cells, 50μL Jurkat cells, and 50μL anti-CD3 antibody per well. Secreted IL-2 was measured on 100μL supernatant using Human IL-2 Quantikine ELISA kit from R&D systems (cat# D2050) according to manufacturer’s recommended protocol. To perform ELISA on IL-10, PEL cells were grown for 24 hours with/without Pom and supernatants were diluted appropriately before performing ELISA using Human IL-10 Quantikine ELISA kit from R&D systems (cat# D1000B).

### NK cell-mediated cytotoxicity assay

NK-mediated cytotoxicity was assessed in the absence of ADCC (antibody-dependent cell-mediated cytotoxicity)-inducing antibody as previously described [29]. Briefly, the YTS NK cell line was used as the effector NK cells and cytotoxicity was assessed using a two-color fluorescence assay. PEL cells (3x10^5^ per mL) cultured in the absence or presence of Pom for 2 days were suspended at 2x10^6^ cells per mL with complete media. NK cells were suspended at 10^6^ cells per mL in PBS and stained with a green fluorescent membrane dye DiOC_18_ (DiO) (Sigma) at a 20μM final concentration by incubating for 30 to 60 minutes at 37°C. The DiO-stained NK cells were suspended at 2x10^6^ cells per mL with complete media after three washes with PBS and then co-cultured with PEL cells at various effector to target ratios by keeping the number of NK cells constant at 10^5^ cells per well of a 48-well plate. After incubating for approximately 3.5 hours at 37°C, propidium iodide (PI) (Sigma) was added at a 75μM final concentration to label dead cells and the cells were analyzed by flow cytometry in the FL1 and FL3 channels for DiO and PI respectively. The percentage of dead target cells was calculated as (DiO-ve, PI+ve cells) x 100 / (total DiO -ve cells). NK-mediated cytotoxicity was calculated as an increase in % dead target cells by subtracting the % dead target cells obtained in the absence of NK cells from that obtained in the presence of NK cells.

### Statistical analysis

Statistical analysis was performed using two-tailed student’s *t-test* (paired or unpaired where indicated) on experiments with at least 3 biological replicates. *P*-values less or equal to 0.05 were considered statistically significant.

## Supporting information

S1 Table Secreted IL2 levels from Jurkat-T cells co-incubated with control or Pom-treated BCBL-1 or JSC-1 cells. Data shows IL2 (pg/mL) present in the supernatant as measured by ELISA after 24 hours co-incubation from 3 independent experiments.
(XLSX)

S1 Fig ICAM-1 and B7-2 surface expression levels and T-cell activation by PEL cell lines compared to non-PEL cell lines. (A) ICAM-1 (red) and B7-2 (blue) levels on the surface of KSHV-infected PEL cell lines BCBL-1, JSC-1, and BC-3, a virus-negative lymphoma line MC116, and EBV-infected BL cell lines Raji and Daudi, as measured by flow cytometry using FITC-conjugated anti-ICAM1 or anti-B7-2 antibodies. (B) Activation of IL2-Jurkat T-cell line, expressed as relative light units (RLU), after costimulation by various lymphomas at a 5:1 T-cell to target ratio in the presence of 2.5 or 10 μg/mL anti-CD3 antibody. Data is presented as an average from at least 3 independent experiments except for MC116 line that was tested only once.
(TIF)

S2 Fig Effect of Pom on the growth of PEL cell lines. BCBL-1, JSC-1, and BC-2 cells were cultured in the absence or presence of indicated concentrations of Pom. After 48 hours (BCBL-1 and JSC-1) or 72 hours (BC-2), live/dead analysis was performed using trypan blue staining. Number of live cells and % viability (% of total cells that are alive) for BCBL-1 (A), JSC-1 (B), and BC-2 (C) were calculated and presented as % of control-treated cells.
(TIF)

S3 Fig Validation of ICAM-1 and B7-2 blocking antibodies. (A and B) BCBL-1 cells grown for 48 hours in the absence or presence of Pom were treated with blocking antibodies (isotype control, anti-ICAM-1 Ab, anti-B7-2 Ab, or both anti-ICAM-1 and anti-B7-2) at a 10ug/mL final concentration each for 30 minutes. The cells were then stained with PE-conjugated anti-ICAM-1 or anti-B7-2 antibodies and flow cytometry was performed to measure the surface expression of ICAM-1 (A) and B7-2 (B). (C) Burkitt’s lymphoma cell line Daudi was pretreated with isotype control or blocking antibodies like in (A) and (B) and then co-incubated for 6 hours with IL2-Jurkat T cells in the presence of 2.5μg/mL anti CD3 antibody. T-cell activation induced by Daudi cells is presented as relative light units (RLU).
(TIF)

S4 Fig Pom does not induce CD80/B7-1 or PD-L1 surface expression in PEL cell lines or CD3 and CD28 in Jurkat T-cells. (A) Surface expression levels of CD80/B7-1 in BCBL-1 or JSC-1 cells treated with indicated concentrations of Pom for 48 hours as measured by flow cytometry using PerCP/Cy5.5-conjugated anti-B7-1 antibody. (B) Surface expression of CD3 and CD28 on Jurkat T cells after 48 hours treatment with Pom measured by flow cytometry using FITC-conjugated anti-CD3 or anti-CD28 antibodies.
(TIF)

S5 Fig Pom-treatment of both PEL and T cells produces higher T-cell activation than treatment of either alone. (A) IL2-Jurkat cells were grown in the absence or presence of 1μM Pom. After 48 hours, Pom was washed out and they were incubated for 6 hours with or without control PEL cells in the absence or presence of various concentrations of anti-CD3 antibody. Activations of ctrl or Pom-treated IL2-Jurkat cells in the absence or presence of ctrl BCBL-1 (left) or JSC-1 (right) cells are expressed as average RLU from 3 separate experiments. (B) Both PEL cells and IL2-Jurkat cells were cultured in the absence or presence of Pom. After 48 hours, cells were washed with PBS to remove Pom and Jurkat T-cell activation assay was performed with various concentrations of anti-CD3 Ab. Activations of ctrl or 1μM Pom-treated Jurkat cells by ctrl or Pom-treated BCBL-1 (left) or JSC-1 (right) cells are expressed as average RLU from 3 separate experiments.
(TIF)

S6 Fig Inhibition of IKZF1, IRF4, and cMyc is not sufficient for Pom-induced increases in surface markers. (A) Relative number of live BCBL-1 cells after 48 hours treatment with indicated concentrations of JQ-1 as measured by trypan blue exclusion method. (B) Protein levels of Ikaros, IRF4, cMyc, and control β-actin in the nuclear lysates of BCBL-1 cells treated with various concentrations of JQ-1 for 48 hours. (C) Surface expression levels of ICAM-1 and B7-2 in BCBL-1 cells 48 hours after treatment with JQ-1. Cells were stained with FITC-conjugated IgG isotype control, anti-ICAM-1, or anti-B7-2 antibodies and analyzed using flow cytometry.
(TIF)

S7 Fig Determining the role of IL-10 signaling in pom-induced increase in ICAM-1 and B7-2. (A) Relative IL-10 levels in the supernatant of BCBL-1 and JSC-1 cells as measured by ELISA 24 hours post treatment with Pom from 3 separate experiments. Average IL-10 produced was ~ 4 ng/mL and ~25 ng/mL in control BCBL-1 and JSC-1 cells respectively. (B) Relative mRNA levels of MARCH 8 and 1 in BCBL-1 cells and MARCH 8 in JSC-1 cells after 48 hours with or without Pom. MARCH 1 was measured but was undetectable in JSC-1 cells. mRNA levels are normalized to that of 18S RNA and expressed as fold change over 0μM-pom treated cells. Error bars represent standard deviation from 3 independent experiments. (C) Surface expression level of CD83 48 hours post-treatment with DMSO ctrl or Pom. (D and E) BCBL-1 cells were pretreated with anti IL-10 Ab (10μg/mL) or recombinant human IL-10 (rIL-10) (100ng/mL) for approximately 1 hour and then treated with 1μM Pom for another 48 hours. Surface expression levels of ICAM-1 and B7-2 were then measured by flow cytometry after staining the cells with PerCP/Cy5.5-conjugated antibodies. Histograms show ICAM-1 and B7-2 levels in the presence of anti-IL-10 Ab and/or Pom (D) or in the presence of rIL-10 and/or Pom (E).
(TIF)

S8 Fig Determining the role of STAT-3 signaling in pom-induced increases in ICAM-1 and B7-2. (A) Surface expression levels of ICAM-1 and B7-2 in BCBL-1 cells 48 hours after treatment with DMSO ctrl or STAT3 inhibitor S3I-201 (10 and 100μM). (B) BCBL-1 cells were pretreated with S3I-201 for approximately 1 hour and then treated with 1μM Pom for another 48 hours. Histograms show ICAM-1 and B7-2 levels in the presence of 1μM Pom alone or in combination with S3I-201. Cells were stained with PerCP/Cy5.5-conjugated antibodies and analyzed using flow cytometry.
(TIF)
