# Supplementary figures and images for: Pomalidomide restores immune recognition of primary effusion lymphoma through upregulation of ICAM-1 and B7-2
Source: PLoS Pathog. 2021 Jan 7;17(1):e1009091. doi: 10.1371/journal.ppat.1009091 (PMC7817053; doi:10.1371/journal.ppat.1009091)

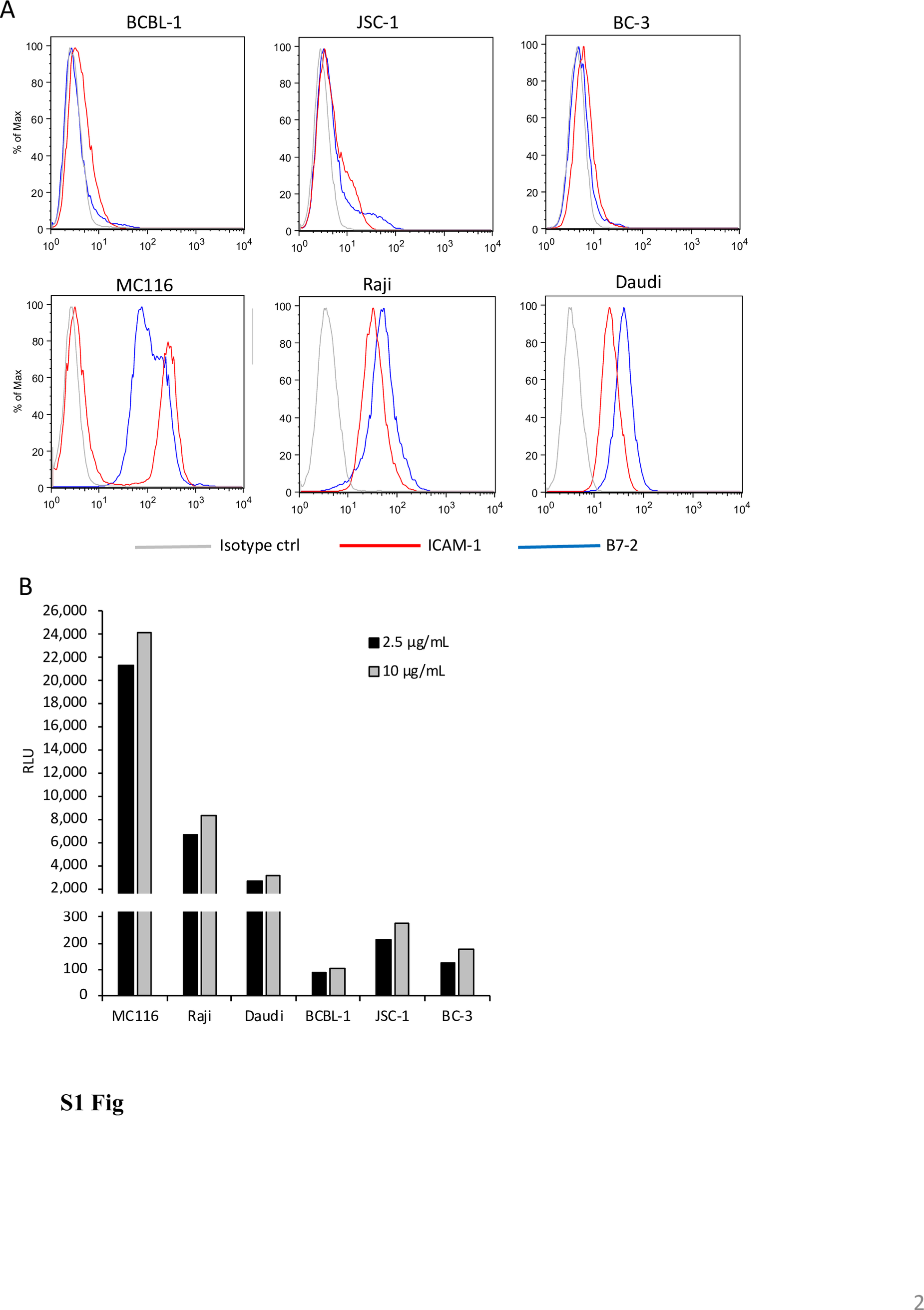

Supplement: S1 Fig — (A) ICAM-1 (red) and B7-2 (blue) levels on the surface of KSHV-infected PEL cell lines BCBL-1, JSC-1, and BC-3, a virus-negative lymphoma line MC116, and EBV-infected BL cell lines Raji and Daudi, as measured by flow cytometry using FITC-conjugated anti-ICAM1 or anti-B7-2 antibodies. (B) Activation of IL2-Jurkat T-cell line, expressed as relative light units (RLU), after costimulation by various lymphomas at a 5:1 T-cell to target ratio in the presence of 2.5 or 10 μg/mL anti-CD3 antibody. Data is presented as an average from at least 3 independent experiments except for MC116 line that was tested only once. (TIF) [file ppat.1009091.s002.tif]

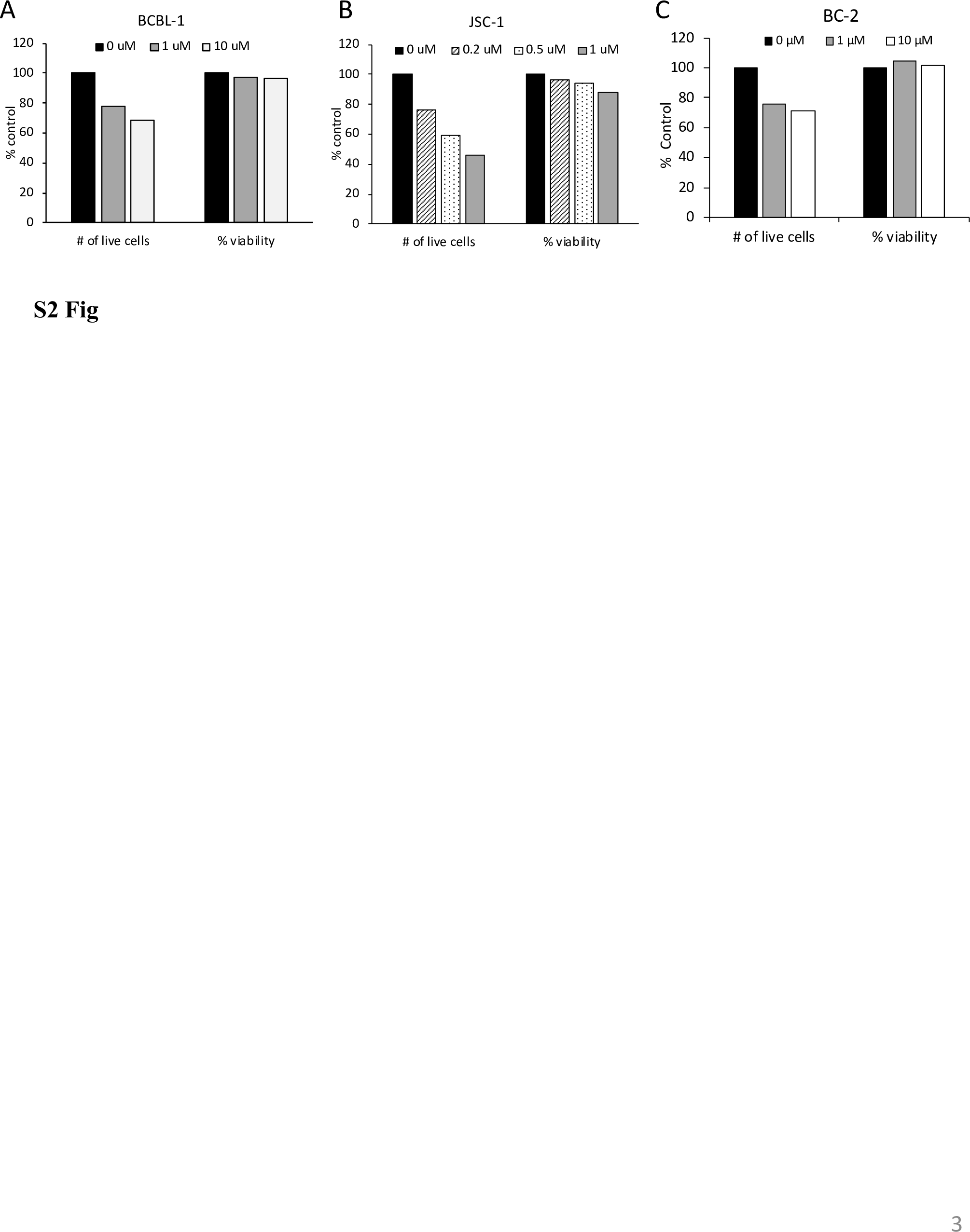

Supplement: S2 Fig — BCBL-1, JSC-1, and BC-2 cells were cultured in the absence or presence of indicated concentrations of Pom. After 48 hours (BCBL-1 and JSC-1) or 72 hours (BC-2), live/dead analysis was performed using trypan blue staining. Number of live cells and % viability (% of total cells that are alive) for BCBL-1 (A), JSC-1 (B), and BC-2 (C) were calculated and presented as % of control-treated cells. (TIF) [file ppat.1009091.s003.tif]

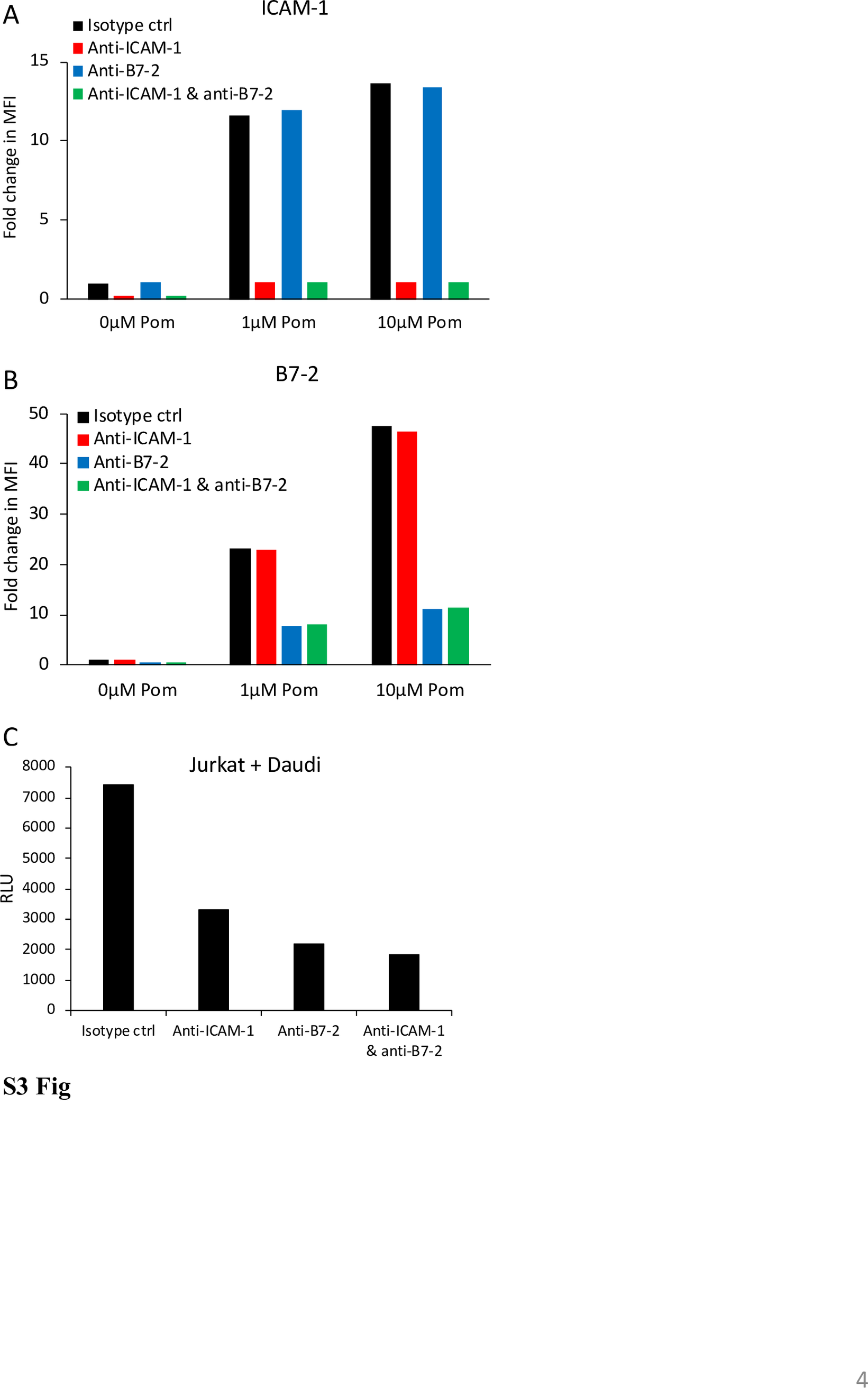

Supplement: S3 Fig — (A and B) BCBL-1 cells grown for 48 hours in the absence or presence of Pom were treated with blocking antibodies (isotype control, anti-ICAM-1 Ab, anti-B7-2 Ab, or both anti-ICAM-1 and anti-B7-2) at a 10ug/mL final concentration each for 30 minutes. The cells were then stained with PE-conjugated anti-ICAM-1 or anti-B7-2 antibodies and flow cytometry was performed to measure the surface expression of ICAM-1 (A) and B7-2 (B). (C) Burkitt’s lymphoma cell line Daudi was pretreated with isotype control or blocking antibodies like in (A) and (B) and then co-incubated for 6 hours with IL2-Jurkat T cells in the presence of 2.5μg/mL anti CD3 antibody. T-cell activation induced by Daudi cells is presented as relative light units (RLU). (TIF) [file ppat.1009091.s004.tif]

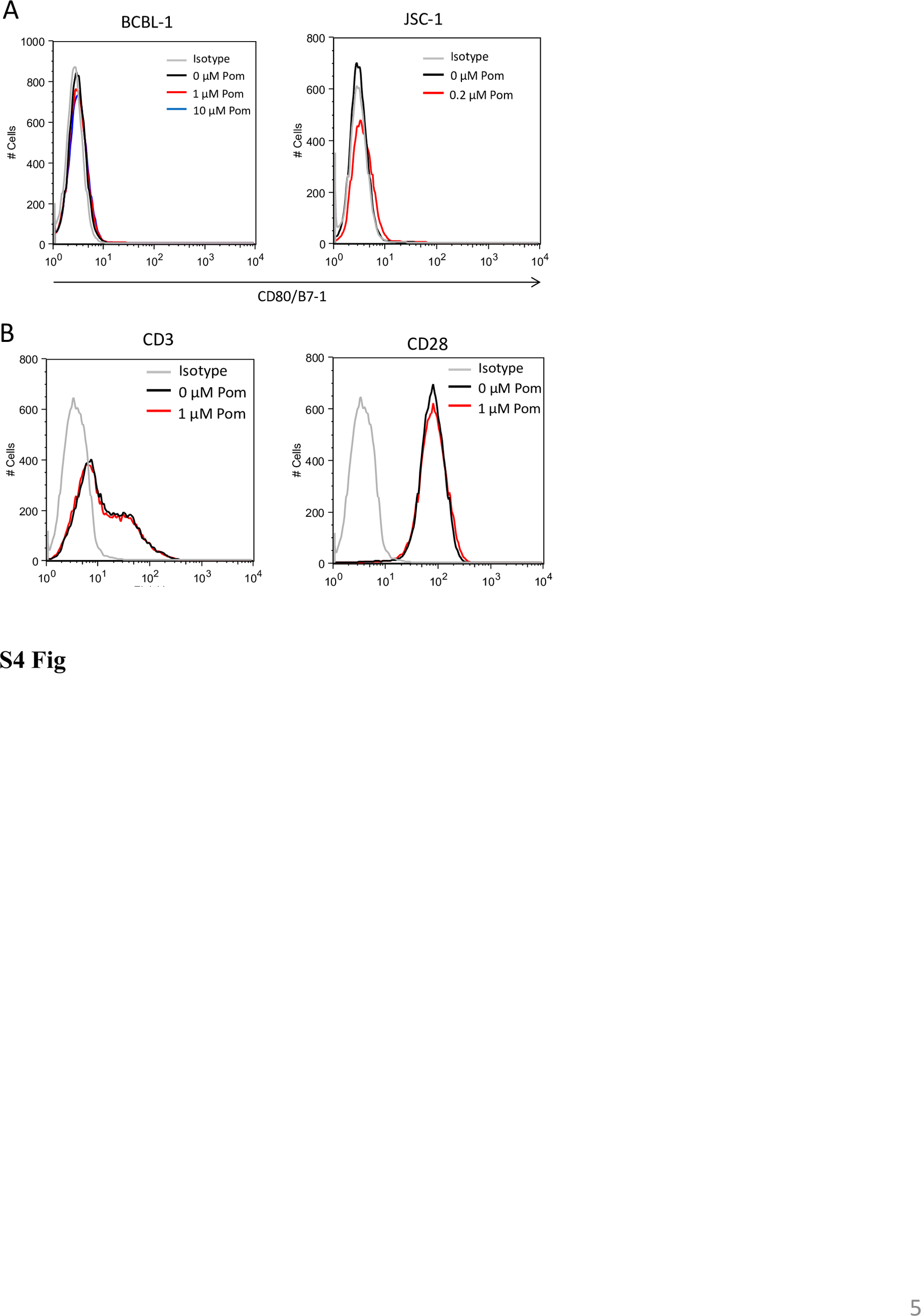

Supplement: S4 Fig — (A) Surface expression levels of CD80/B7-1 in BCBL-1 or JSC-1 cells treated with indicated concentrations of Pom for 48 hours as measured by flow cytometry using PerCP/Cy5.5-conjugated anti-B7-1 antibody. (B) Surface expression of CD3 and CD28 on Jurkat T cells after 48 hours treatment with Pom measured by flow cytometry using FITC-conjugated anti-CD3 or anti-CD28 antibodies. (TIF) [file ppat.1009091.s005.tif]

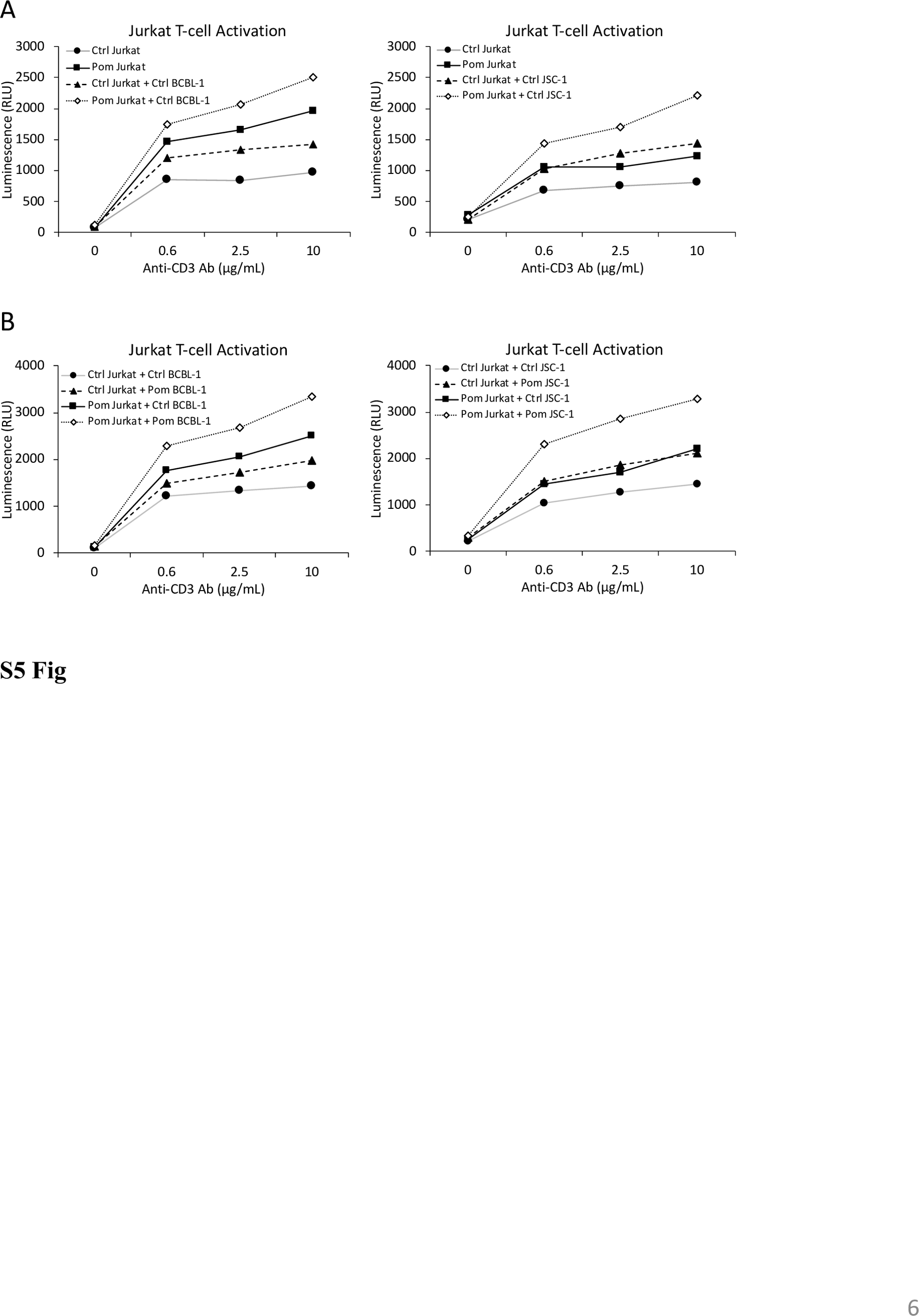

Supplement: S5 Fig — (A) IL2-Jurkat cells were grown in the absence or presence of 1μM Pom. After 48 hours, Pom was washed out and they were incubated for 6 hours with or without control PEL cells in the absence or presence of various concentrations of anti-CD3 antibody. Activations of ctrl or Pom-treated IL2-Jurkat cells in the absence or presence of ctrl BCBL-1 (left) or JSC-1 (right) cells are expressed as average RLU from 3 separate experiments. (B) Both PEL cells and IL2-Jurkat cells were cultured in the absence or presence of Pom. After 48 hours, cells were washed with PBS to remove Pom and Jurkat T-cell activation assay was performed with various concentrations of anti-CD3 Ab. Activations of ctrl or 1μM Pom-treated Jurkat cells by ctrl or Pom-treated BCBL-1 (left) or JSC-1 (right) cells are expressed as average RLU from 3 separate experiments. (TIF) [file ppat.1009091.s006.tif]

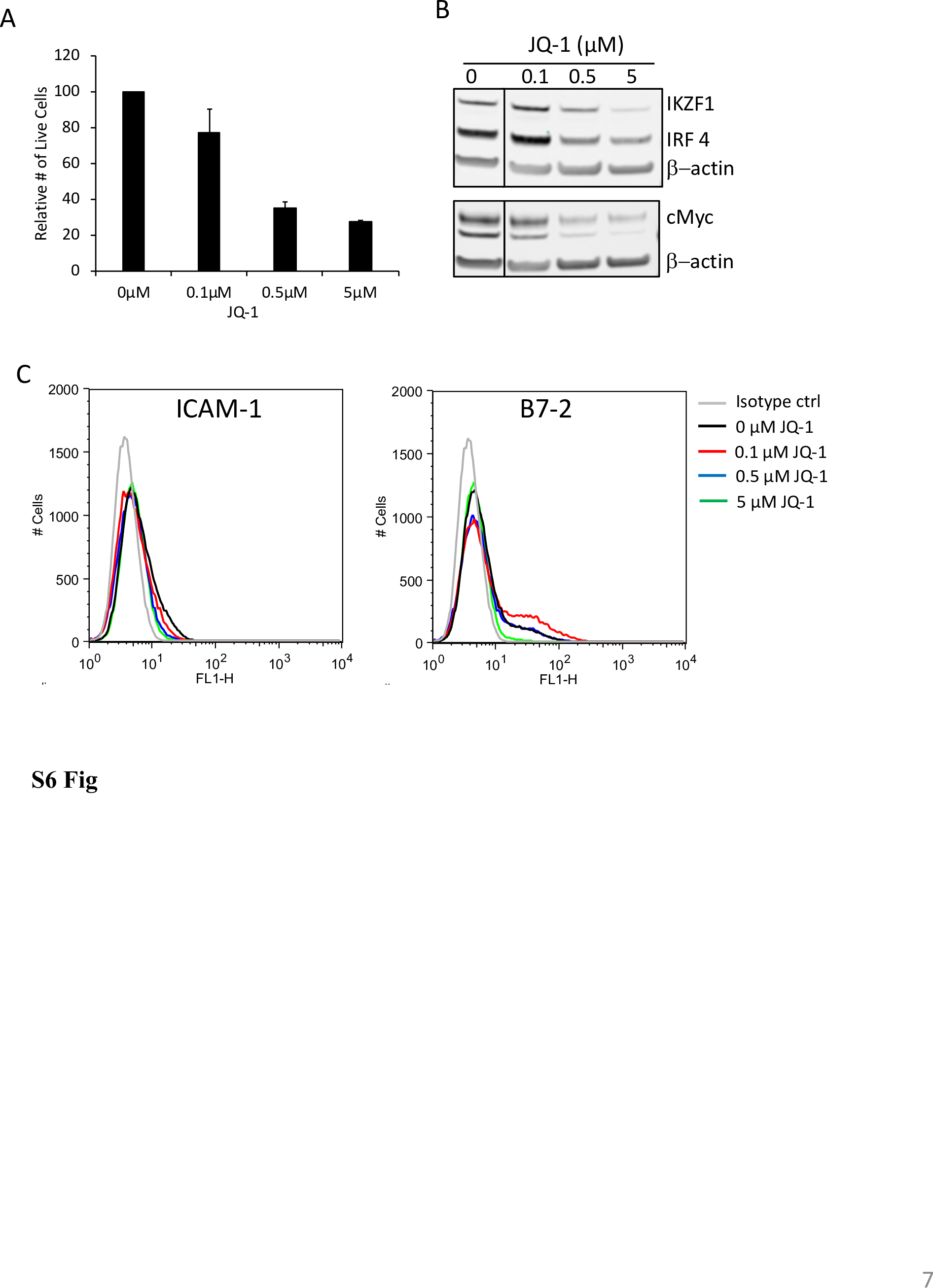

Supplement: S6 Fig — (A) Relative number of live BCBL-1 cells after 48 hours treatment with indicated concentrations of JQ-1 as measured by trypan blue exclusion method. (B) Protein levels of Ikaros, IRF4, cMyc, and control β-actin in the nuclear lysates of BCBL-1 cells treated with various concentrations of JQ-1 for 48 hours. (C) Surface expression levels of ICAM-1 and B7-2 in BCBL-1 cells 48 hours after treatment with JQ-1. Cells were stained with FITC-conjugated IgG isotype control, anti-ICAM-1, or anti-B7-2 antibodies and analyzed using flow cytometry. (TIF) [file ppat.1009091.s007.tif]

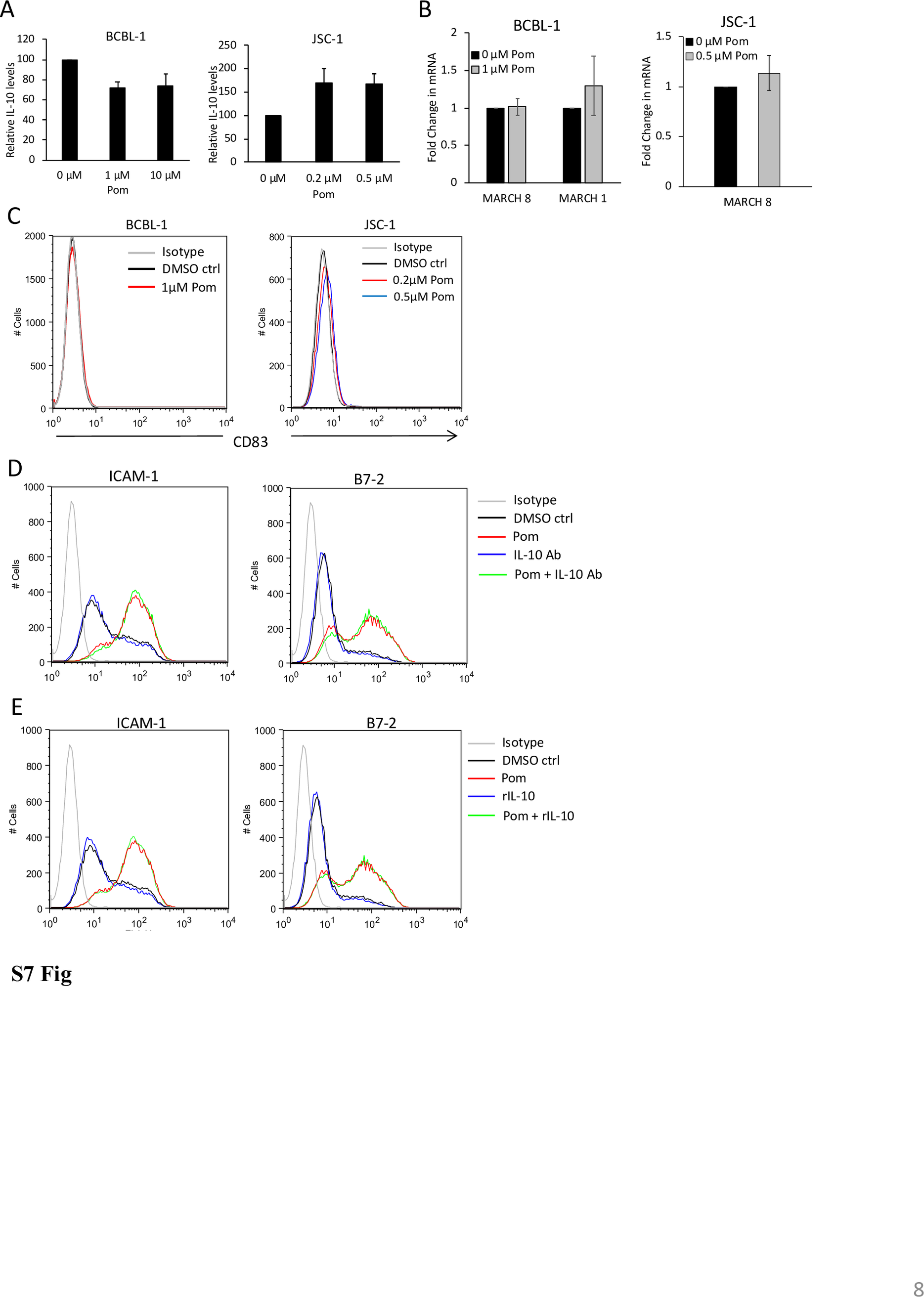

Supplement: S7 Fig — (A) Relative IL-10 levels in the supernatant of BCBL-1 and JSC-1 cells as measured by ELISA 24 hours post treatment with Pom from 3 separate experiments. Average IL-10 produced was ~ 4 ng/mL and ~25 ng/mL in control BCBL-1 and JSC-1 cells respectively. (B) Relative mRNA levels of MARCH 8 and 1 in BCBL-1 cells and MARCH 8 in JSC-1 cells after 48 hours with or without Pom. MARCH 1 was measured but was undetectable in JSC-1 cells. mRNA levels are normalized to that of 18S RNA and expressed as fold change over 0μM-pom treated cells. Error bars represent standard deviation from 3 independent experiments. (C) Surface expression level of CD83 48 hours post-treatment with DMSO ctrl or Pom. (D and E) BCBL-1 cells were pretreated with anti IL-10 Ab (10μg/mL) or recombinant human IL-10 (rIL-10) (100ng/mL) for approximately 1 hour and then treated with 1μM Pom for another 48 hours. Surface expression levels of ICAM-1 and B7-2 were then measured by flow cytometry after staining the cells with PerCP/Cy5.5-conjugated antibodies. Histograms show ICAM-1 and B7-2 levels in the presence of anti-IL-10 Ab and/or Pom (D) or in the presence of rIL-10 and/or Pom (E). (TIF) [file ppat.1009091.s008.tif]

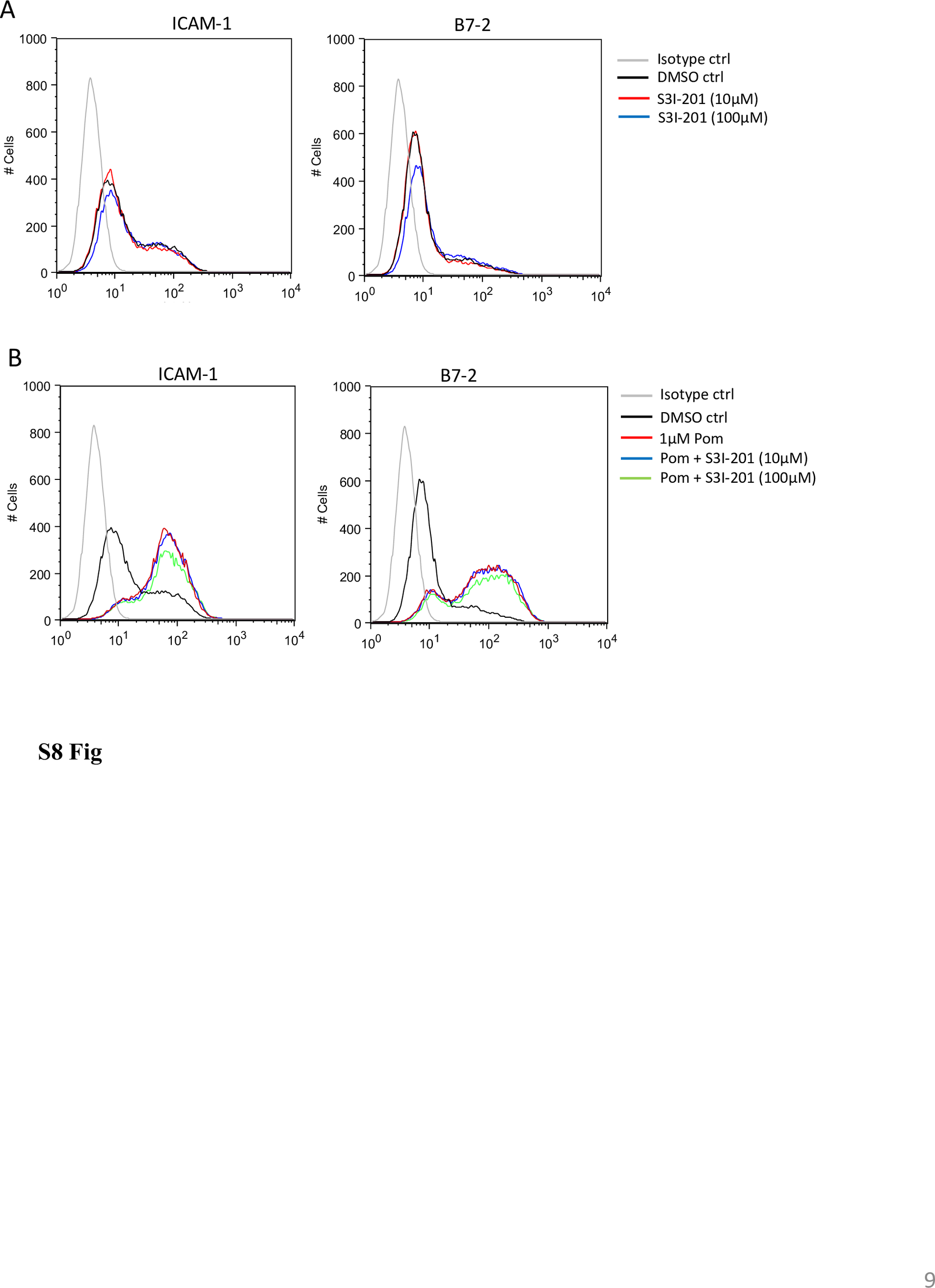

Supplement: S8 Fig — (A) Surface expression levels of ICAM-1 and B7-2 in BCBL-1 cells 48 hours after treatment with DMSO ctrl or STAT3 inhibitor S3I-201 (10 and 100μM). (B) BCBL-1 cells were pretreated with S3I-201 for approximately 1 hour and then treated with 1μM Pom for another 48 hours. Histograms show ICAM-1 and B7-2 levels in the presence of 1μM Pom alone or in combination with S3I-201. Cells were stained with PerCP/Cy5.5-conjugated antibodies and analyzed using flow cytometry. (TIF) [file ppat.1009091.s009.tif]
